# The tRNA moieties of both aminoacyl-tRNA substrates of a cyclodipeptide synthase share a common binding site, as revealed by RNA microhelices mimicking tRNA acceptor arms

**DOI:** 10.1093/nar/gkag307

**Published:** 2026-04-07

**Authors:** Zahra Marouf, Carine Tellier-Lebegue, Matthieu Glousieau, Nelly Morellet, Philippe Cuniasse, Alexa Bourand-Plantefol, Stéphane Plancqueel, Ikram Mahmoudi, Jessica Andreani, Pierre Legrand, Virginie Ropars, Rémi Ruedas, Léa-Fleur Hermouet, Mireille Moutiez, Naïma Nhiri, Matthieu Fonvielle, Stéphane Bressanelli, Takayuki Katoh, Paloma Fernandez Varela, Ewen Lescop, Jean-Baptiste Charbonnier, Muriel Gondry

**Affiliations:** Institute for Integrative Biology of the Cell (I2BC), Université Paris-Saclay, CEA, CNRS, Gif-sur-Yvette, France; Institut de Chimie des Substances Naturelles, CNRS UPR 2301, Université Paris-Saclay, Gif-sur-Yvette, France; Institute for Integrative Biology of the Cell (I2BC), Université Paris-Saclay, CEA, CNRS, Gif-sur-Yvette, France; Institute for Integrative Biology of the Cell (I2BC), Université Paris-Saclay, CEA, CNRS, Gif-sur-Yvette, France; Institut de Chimie des Substances Naturelles, CNRS UPR 2301, Université Paris-Saclay, Gif-sur-Yvette, France; Institute for Integrative Biology of the Cell (I2BC), Université Paris-Saclay, CEA, CNRS, Gif-sur-Yvette, France; Institute for Integrative Biology of the Cell (I2BC), Université Paris-Saclay, CEA, CNRS, Gif-sur-Yvette, France; Institute for Integrative Biology of the Cell (I2BC), Université Paris-Saclay, CEA, CNRS, Gif-sur-Yvette, France; Institute for Integrative Biology of the Cell (I2BC), Université Paris-Saclay, CEA, CNRS, Gif-sur-Yvette, France; Institute for Integrative Biology of the Cell (I2BC), Université Paris-Saclay, CEA, CNRS, Gif-sur-Yvette, France; Synchrotron SOLEIL, Saint Aubin, France; Institute for Integrative Biology of the Cell (I2BC), Université Paris-Saclay, CEA, CNRS, Gif-sur-Yvette, France; Institute for Integrative Biology of the Cell (I2BC), Université Paris-Saclay, CEA, CNRS, Gif-sur-Yvette, France; Institute for Integrative Biology of the Cell (I2BC), Université Paris-Saclay, CEA, CNRS, Gif-sur-Yvette, France; Institute for Integrative Biology of the Cell (I2BC), Université Paris-Saclay, CEA, CNRS, Gif-sur-Yvette, France; Institut de Chimie des Substances Naturelles, CNRS UPR 2301, Université Paris-Saclay, Gif-sur-Yvette, France; Institute for Integrative Biology of the Cell (I2BC), Université Paris-Saclay, CEA, CNRS, Gif-sur-Yvette, France; Institute for Integrative Biology of the Cell (I2BC), Université Paris-Saclay, CEA, CNRS, Gif-sur-Yvette, France; Department of Chemistry and Biotechnology, Graduate School of Engineering, The University of Tokyo, Tokyo, Japan; Institute for Integrative Biology of the Cell (I2BC), Université Paris-Saclay, CEA, CNRS, Gif-sur-Yvette, France; Institut de Chimie des Substances Naturelles, CNRS UPR 2301, Université Paris-Saclay, Gif-sur-Yvette, France; Institute for Integrative Biology of the Cell (I2BC), Université Paris-Saclay, CEA, CNRS, Gif-sur-Yvette, France; Institute for Integrative Biology of the Cell (I2BC), Université Paris-Saclay, CEA, CNRS, Gif-sur-Yvette, France

## Abstract

Cyclodipeptide synthases (CDPSs) sequentially use two aminoacyl-tRNAs (AA-tRNAs) as substrates to catalyze cyclodipeptide formation. We previously showed that microhelices (miHxs), which mimic the tRNAs acceptor arms, are as efficient as full-length AA-tRNAs as substrates when aminoacylated by flexizymes. We generated a diverse set of miHxs (acylated, unacylated, misacylated, mutated, or shortened miHxs) and analyzed their interactions with CDPSs. We studied the *Nocardia brasiliensis* CDPS (*Nbra*-CDPS), which synthesizes cyclo(l-Ala-l-Glu) using Ala-tRNA^Ala^ and Glu-tRNA^Glu^ as its first and second substrates, respectively. We determined the crystal structure of *Nbra*-CDPS bound to two analogues of its first substrate, unacylated miHx^Ala^ and acylated miHx^Ala^, in which alanine is attached *via* an amide bond. We showed by cryoEM that the miHx^Ala^ mimics well the acceptor stem of the full-length tRNA^Ala^. We determined the crystal structure of *Nbra*-CDPS bound to unacylated miHx^Glu^, an analogue of its second substrate, and showed that, despite sequence differences, it superimposes well with miHx^Ala^. This result, combined with the use of misacylated substrates, indicates that the RNA stem moieties of both substrates share a common binding mode. Together, our findings establish miHxs as powerful tools for dissecting CDPS substrate recognition and provide a framework for studying other AA-tRNA-utilizing enzymes.

## Graphical Abstract

**Graphical Abstract:**
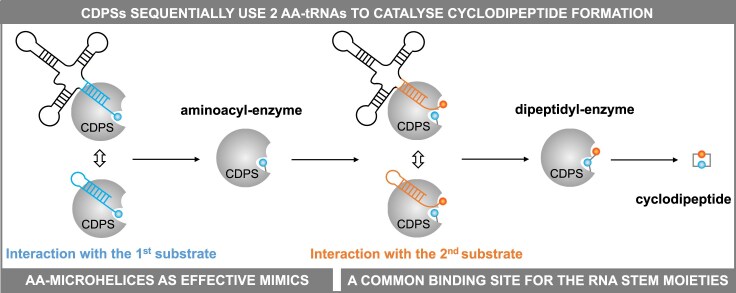

## Introduction

Cyclodipeptide synthases (CDPSs) are a family of tRNA-dependent enzymes involved in the biosynthesis of cyclodipeptides [1–3]. These enzymes are frequently associated with tailoring enzymes that modify cyclodipeptides, giving rise to a large ensemble of specialized metabolites known as 2,5-diketopiperazines (2,5-DKPs) [1, 4, 5]. Many 2,5-DKPs display relevant biological activities, including antibacterial, antifungal, antiviral, antiprion, and antitumoral properties [6–8]. CDPSs divert aminoacyl-tRNAs (AA-tRNAs) from their canonical role in ribosomal translation to catalyze two peptide-bond-forming reactions, resulting in the synthesis of cyclodipeptides [2, 9]. The amino acid composition of cyclodipeptides is defined by the specific AA-tRNAs recruited in sequence by the enzyme. More than 120 CDPSs have been characterized for their cyclodipeptide-synthesizing activity, collectively producing over 80 of the 210 possible natural cyclodipeptides [1]. In addition, CDPSs can accept non-canonical amino acids, enabling the synthesis of ∼200 unnatural 2,5-DKPs [10]. Investigating the molecular bases of CDPSs substrate specificity, i.e. understanding the interactions between CDPSs and their AA-tRNA substrates, is essential for engineering enzymes with broadened specificities.

**Figure 1.**
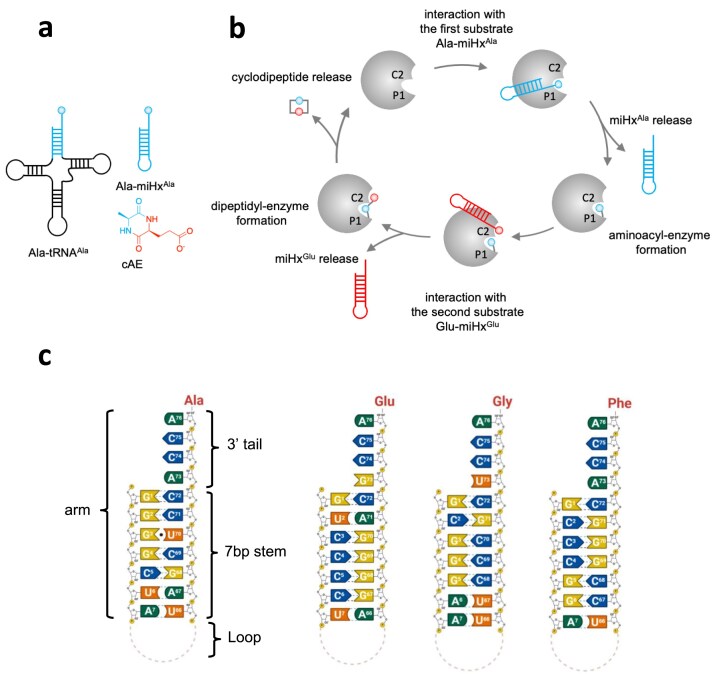
Investigation of the interaction between *Nbra*-CDPS and AA-miHxs. (a) Substrates and product of *Nbra*-CDPS: the first substrate used, Ala-miHx^Ala^, an analogue mimicking the acceptor arm of Ala-tRNA^Ala^ (shown in blue); the resulting product, cAE, with a blue moiety derived from the first substrate and a red moiety derived from the second. (b) Schematic representation of the catalytic cycle of *Nbra*-CDPS. The enzyme first interacts with Ala-miHx^Ala^, whose aminoacyl moiety is accommodated in the P1 pocket and transferred to the catalytic serine at the pocket entrance, leading to the formation of an alanyl-enzyme intermediate. Glu-miHx^Glu^ then interacts with the enzyme, with its glutamyl moiety accommodated in a cleft named C2, and is transferred to form a dipeptidyl-enzyme intermediate. This intermediate undergoes intramolecular cyclization, resulting in the release of cAE. (c) Sequences of the miHxs used (numbering corresponds to tRNAs, and nucleotides are color-coded by base identity). The loop region introduced to stabilize the miHx is 5′-U^8^UCG^11^. Created in BioRender, Marouf, Z. (2026) https://BioRender.com/vzvcs7q.

CDPSs are currently divided into three subfamilies: the NYH and XYP, identified based on sequence homology, and the recently discovered RCDPS subfamily, which shows no sequence homology with the other two [11, 12]. Both NYH and XYP subfamilies have been well studied [1, 3]. They share a conserved catalytic mechanism involving four residues (S37, Y178, E182, Y202; residue numbering based on the CDPS from *Streptomyces noursei*, called *Snou*-CDPS) but differ mostly in a key pair of residues located in the active site region: N40-H203 in NYH CDPSs versus X40-P203 in XYP enzymes where X can be any amino acid [3, 11, 13–17]. The catalytic cycle begins with the binding of the first AA-tRNA in such a way that the aminoacyl moiety is positioned in the pocket called P1 (Fig. 1a,b and SI Fig. 1) and then transferred to the conserved serine (S37 in *Snou*-CDPS numbering), forming an aminoacyl-enzyme intermediate. The second AA-tRNA then interacts with the intermediate such as that its aminoacyl moiety, accommodated in a cleft referred to as C2 (previously called P2 pocket), is transferred to the aminoacyl-enzyme, generating a dipeptidyl-enzyme intermediate (Fig. 1b and SI Fig. 1). Finally, the dipeptidyl moiety undergoes an intramolecular cyclization involving a conserved tyrosine residue (Y202 in *Snou*-CDPS numbering), leading to the final cyclodipeptide.

To date, nine CDPSs crystal structures have been reported in their substrate-free forms (including five from the NYH family and four from the XYP family) [15, 18–20]. CDPSs belong to the HUP domain superfamily, defined by Aravind *et al.* [21], which also includes class I aminoacyl-tRNA synthetases (AARSs) and enzymes involved in NAD and FAD biosynthesis. A crystal structure of the NYH *Snou-*CDPS covalently attached to a dipeptide analogue corresponding to the second reaction intermediate state was reported [16]. It allowed to define the pocket P1 and the cleft C2 mentioned above (SI Fig. 1). Recently, the crystal structure of the XYP CDPS from *Candidatus Glomeribacter gigasporarum* (*Cglo*-CDPS) was determined at 5 Å resolution in complex with its first Phe-tRNA^Phe^ substrate [22]. It showed that *Cglo*-CDPS engages exclusively with the substrate’s acceptor arm *via* two basic β-strands, which interact with the major groove of the acceptor stem. The 3′-ACCA extremity adopts a direction that deviates from the helix axis of the acceptor stem, positioning the phenylalanyl moiety toward the P1 pocket. Clearly, structural studies are limited by resolution, primarily due to tRNA flexibility (as seen with *Cglo*-CDPS, PDB: 6Y4B for *Cglo*-CDPS^S32A^:Phe-tRNA^Phe^ complex), which remains a major bottleneck for characterizing CDPS-substrate interactions at atomic detail.

In our study, we investigated the molecular basis of the interaction between CDPSs and the tRNA moieties of their two substrates, keeping in mind that the binding of the first substrate still needs to be characterized more precisely and that the binding mode of the second substrate remains to be elucidated. As a model, we studied the XYP CDPS from *Nocardia brasiliensis* (*Nbra*-CDPS), which synthesizes the heterocyclodipeptide cyclo(l-Ala-l-Glu) (cAE) predominantly from Ala-tRNA^Ala^ as its first substrate and Glu-tRNA^Glu^ as its second substrate (Fig. 1a,b). *Nbra*-CDPS produces minor amounts of homocyclodipeptides (2% cAA and no cEE) [9, 11]. As most CDPSs produce homocyclodipeptides, previous studies focused on enzymes that use the same two AA-tRNAs, making it difficult to discriminate their respective binding sites. We recently showed that the acceptor arms of the two substrates are the only parts of the tRNAs needed for *Nbra*-CDPS enzymatic activity [9]. We therefore investigate the interactions of *Nbra*-CDPS with the two microhelices (miHxs), miHx^Ala^ or miHx^Glu^, which mimic the acceptor arms of tRNA^Ala^ and tRNA^Glu^, respectively (Fig. 1). We also investigated the interactions and enzymatic activity of this enzyme in the presence of acylated miHxs and various analogues, which were prepared using flexizymes (Fxs), as these are versatile RNA acylation catalysts (SI Fig. 2) [23]. No crystal structure of CDPS complexes is available with full miHxs and other AA-tRNA-dependent enzymes to evaluate how miHxs can mimic tRNA acceptor stems. The promising properties of miHxs, including their increased rigidity and smaller size (compared to tRNA), as well as their ability to be acylated or misacylated with any amino acids, convinced us to use them as tRNA mimics for structural, biophysical, and catalytic activity measurements.

Here, we present three crystal structures of *Nbra*-CDPS in complex with miHxs. Two structures show the enzyme bound to analogues of its first substrate: a stable Ala-(amide)-miHx^Ala^ acylated with alanine *via* an amide bond instead of an ester bond (structure obtained at 3.6 Å resolution) and an unacylated miHx^Ala^ (3.3 Å resolution). The third structure shows the enzyme bound to an analogue of its second substrate, an unacylated miHx^Glu^ (3.4 Å resolution). These structures revealed the conformation of the 3′ tails within the *Nbra*-CDPS active site and the sequence-specific interactions between *Nbra*-CDPS and the helical stems of miHxs. Cryo-EM confirmed that miHx^Ala^ effectively occupies the same position and orientation as the acceptor stem of a full-length tRNA^Ala^, and modelling of the step immediately following Ala-miHx^Ala^ binding reveals the structure of the aminoacyl-enzyme intermediate. Biophysical and enzymatic assays, with unacylated, acylated, misacylated, and mutated miHxs, allowed us to define the key interaction features between *Nbra*-CDPS and the acceptor arms of both substrates. They also further identified key structural features critical for catalysis.

## Material and methods

### Construction, expression, and purification of *Nbra*-CDPS and its variants

For overexpression in *Escherichia coli* of *Nbra*-CDPS, the gene encoding *Nocardia brasiliensis* CDPS (*Nbra*-CDPS) fused to a *C*-terminal His-Tag was cloned into the PIJ196 vector as previously described [11]. Hereafter, “*Nbra*-CDPS” refers to the dimeric wild-type *Nbra*-CDPS, while a monomeric variant, named *Nbra*-CDPS*^mono^*, was also used in our study (see below). Site-directed mutagenesis was performed by PCR using the QuikChange method (Stratagene) with primers listed in SI Table 1 to generate the following variants: *Nbra*-CDPS^S34A^, in which the catalytic serine 34 (equivalent to S37 in *Snou*-CDPS) was replaced with alanine, *Nbra*-CDPS^L32A^, *Nbra*-CDPS^L32G^, and *Nbra*-CDPS^S199L^. A *Nbra*-CDPS^mono^ was constructed and corresponds to a monomeric variant of the dimeric wild-type enzyme by introducing the N108E, F114D, and S117R mutations. The variant *Nbra*-CDPS^mono/S34A^ (the monomeric variant in which S34 is replaced with Ala) was also generated. *Nbra*-CDPS and its variants were purified following a previously established protocol [9]. For the untagged form of *Nbra*-CDPS^S34A^, expression and cell lysis were carried out in the same manner; then the supernatant was purified on a HiTrap Heparin HP affinity column (Cytiva). After dialysis, the protein was further purified on a RESOURCE Q column (Cytiva). The oligomeric state of the dimeric or monomeric enzymes was analyzed by Size Exclusion Chromatography (SEC) on a Superdex 75 10/300 column equilibrated in phosphate buffer pH 7.5, at a flow rate of 0.4 mL/min. The detailed protocol for obtaining ^15^N, ^15^N-^13^C, and ^15^N-^13^C-^2^H *Nbra*-CDPS^mono/S34A^ for NMR studies is provided in the Supp Methods.

### Preparation of miHxs and Ala-tRNA^Ala^

Because *Nbra*-CDPS is active in *E. coli*, we used *E. coli* tRNAs and their corresponding miHxs, as previously described [9, 11]. MiHxs reproduce the full acceptor arms of tRNAs, consisting of a 7 bp stem and a 3′-NCCA tail. Shortened miHxs mutants are indicated with a subscript reflecting the number of base pairs retained (e.g. miHx^Ala-6^ for a 6 bp version starting from the 3′-NCCA tail). MiHxs and their shortened analogues were purchased from Eurogentec except NH_2_-miHx^Ala^, which was prepared for our study as described in [24].

The dFx variant of Fxs was produced as previously described [9]. It acylates the 3′-OH or 3′-NH_2_ group of any RNA molecule that terminates in a 3′-NCCA sequence, using chemically-activated amino acids [24] (SI Fig. 2). The synthesis of dinitrobenzyl esters of l-Ala (Ala-DBE) and l-Glu (Glu-DBE) was carried out according to standard procedures [25, 26]; Glu-DBE was also purchased from Novalix. MiHxs were aminoacylated by dFx according to the procedure described in the Supp Methods. Ala-tRNA^Ala^ was prepared as described previously using AlaRS for the aminoacylation [9].

### Determination of the cyclodipeptide-synthesizing activities of *Nbra*-CDPS and its variants

These activities were determined both *in vivo* and *in vitro*. The *in vivo* assays were performed as previously described [11, 13]. Each protein was overexpressed in *E. coli*; at the end of the cultivation, the culture supernatant was analyzed for its cyclodipeptide content. Cyclodipeptides were detected, identified, and quantified by LC-MS/MS analyses on an Agilent 1100 HPLC coupled via a split system to an Esquire HCT ion-trap mass spectrometer (Bruker Daltonics GmbH) set in positive mode. The detailed procedure is described in the Supp Methods. The cyclodipeptide-synthesizing activities of the variants were determined from three independent experiments. The *in vitro* assay was performed in the presence of purified proteins and aminoacylated miHxs and/or tRNAs. Enzymatic end point tests were conducted as previously described [9], and cyclodipeptides were analyzed by LC-MS/MS (see above). Details are given in the Supp Methods. As optimized and defined previously to facilitate cyclodipeptide detection, the substrate concentrations used in the assays were 600 nM for Ala-miHx^Ala^ and its mutants in the presence of 600 nM Glu-tRNA^Glu^ or 600 nM for Glu-miHx^Glu^ and its mutants in the presence of 1200 nM Ala-tRNA^Ala^ [9]. Enzymatic activities are reported as a percentage of the activity for the wild-type enzyme. Duplicates or triplicates were performed to determine standard errors.

### Interactions of *Nbra*-CDPS with miHxs

Interactions between *Nbra*-CDPS with miHxs were first assessed qualitatively by Electrophoretic Mobility Shift Assay (EMSA) using fluorescently labelled oligonucleotides. Complex formation was monitored with FAM-miHx^Ala^ (10 nM), in which 6-carboxyfluorescein (6-FAM) was attached to the deoxyribose at position 10 within the 5′-U^8^U^9^C^10^G^11^-3′ loop. The fluorescent FAM-miHx^Ala^ was incubated with dimeric *Nbra*-CDPS^S34A^ or monomeric *Nbra*-CDPS^mono/S34A^ (concentration ranging from 39 nM to 10 µM) in a buffer (20 mM sodium phosphate, pH 7.5, 120 mM NaCl, 5% glycerol, 1 mM DTT). The complexes were loaded on a native 0.5x Tris/borate/EDTA polyacrylamide gel. Details are provided in the Supp Methods. Quantitative measurements were performed by Bio-Layer Interferometry (BLI) using a Sartorius Octet® RED96e system (Fremont, CA, USA) and Streptavidin (SA) biosensors. Dimeric *Nbra*-CDPS^S34A^ and monomeric *Nbra*-CDPS^mono/S34A^ were analyzed with several miHxs labelled with biotin deoxyribose in position 10 in the loop. The miHx substrates were immobilized at 30 nM in PBS buffer (20 mM sodium phosphate, pH 7.3, 100 mM NaCl) to obtain a signal of 0.2 nm (Δλ). Association was measured during 300 s in 200 µL of enzyme solution (15.6, 31.3, or 62.5 nM) in PBS-T buffer (20 mM sodium phosphate, pH 7.3, 100 mM NaCl, 0.1% Tween20), followed by dissociation for 600 s in PBS-T without protein. To assess the influence of Tween20 on *Nbra*-CDPS stability, we collected TSA (Thermal Shift Assay) data (SI Fig. 3). All experiments were performed in duplicate. Non-specific signals obtained with blank sensors were subtracted from the functionalized sensorgrams.

### Crystal structures of *Nbra*-CDPS with or without miHxs

Crystallization screenings were performed at the I2BC crystallization platform using a Mosquito-LCP robot (TTP LabTech’s) and a RockImager 1000 (Formulatrix) for crystal visualization. Screenings with NeXtal crystallization 96-well kits (The Classics, The Nucleix, and The PEGs Suites) were performed at 4°C and 18°C. Crystallization assays were performed with *Nbra*-CDPS, inactive *Nbra*-CDPS^S34A^, and untagged inactive *Nbra*-CDPS^S34A^ at concentrations ranging from 1.5 mg/mL to 9 mg/mL, in the presence or absence of a 1.2–6 molar excess of miHx^Ala^. We used low NaCl salt concentrations as well as xylitol to increase the stability of the RNA/protein complexes and to improve the quality of the crystals. These crystallization conditions yielded the *Nbra*-CDPS/miHx^Ala^ complex diffracting to 3.3 Å. These conditions used for *Nbra*-CDPS/miHx^Glu^ complex yielded also two crystal forms of this complex, which diffracted to 3.4 Å and 3.5 Å resolution. Crystals of the untagged apo *Nbra*-CDPS^S34A^ diffracted to 1.7 Å. Diffraction data were collected at 100K on the PROXIMA-2A beamline at the synchrotron SOLEIL. The information on the data collection, structure determination, refinement statistics, and quality of the models for all the crystal structures is presented in the Supp Methods.

We did not succeed in obtaining structural information on the alanylated ester forms of the miHx^Ala^, neither with the *Nbra*-CDPS nor with the *Nbra*-CDPS^S34A^ variant. In parallel, we performed several assays to crystallize *Nbra*-CDPS or the *Nbra*-CDPS^S34A^ variant with the stable Ala-(amide)-miHx^Ala^. We collected diffraction data at 3.6 Å with *Nbra*-CDPS^S34A^ and Ala-(amide)-miHx^Ala^ and observed that these crystals have the same unit cell compared to the *Nbra*-CDPS^S34A^/miHx^Ala^ complex. Diffraction data were collected as previously mentioned.

### Molecular simulation of the structure of the aminoacyl-enzyme intermediate, in which S34 is alanylated

To further investigate the residues of *Nbra*-CDPS susceptible to influencing the specificity of the enzyme for the first residue incorporated into the cyclodipeptide, we performed a molecular modelling study of the enzyme’s alanylated intermediate at residue S34. At this step, the alanyl moiety of Ala-miHx^Ala^ was transferred to S34 to form the aminoacyl-enzyme (SI Fig. 4). The method used for molecular simulation is detailed in the Supp Methods.

### NMR analyses of the interactions between *Nbra*-CDPS^mono/S34A^ and miHx^Ala^ in the presence or absence of MgCl_2_

NMR samples were prepared in a buffer containing 20 mM NaHPO_4_, pH 7.3, 100 mM NaCl, 5% glycerol, 1 mM DTT, and 95% H_2_O / 5% D_2_O. A NOESY experiment with a 160 ms mixing time was recorded on miHx^Ala^ to assign the imino protons. Titrations of 150 μM miHx^Ala^ with 0–8 mM of MgCl_2_ and with 0–2 molar equivalents of *Nbra*-CDPS^mono/S34A^ were followed by solvent-suppressed ^1^H 1D spectra. Spectra were acquired on a 950 MHz AVANCE III HD Bruker spectrometer equipped with a TCI cryoprobe at 283 K. Additional information regarding the NMR analyses is reported in the Supp Methods.

### Cryo-EM structure of *Nbra*-CDPS^S34A^ bound to tRNA^Ala^

Cryo-EM data were collected on a Glacios transmission microscope equipped with a Falcon4i camera on the Nanoimaging platform (Institut Pasteur, Paris). Data collection parameters are provided in the Supp Methods, SI Fig. 12, and SI Table 3. Several rounds of 2D classification were performed, followed by *ab initio* reconstruction and heterogeneous refinement. Final local refinement yielded a reconstruction at 4.2 Å resolution from 209 133 selected particles.

### Multiple sequence alignments and evolutionary conservation mapping

A curated set of annotated XYP sequences [1] was realigned using the MAFFT G-INS-i algorithm [27]. Evolutionary conservation scores were then computed using the Rate4Site algorithm as implemented on the Consurf web server [28].

## Results

### Characterization of the dimeric and monomeric forms of *Nbra*-CDPS

**Figure 2.**
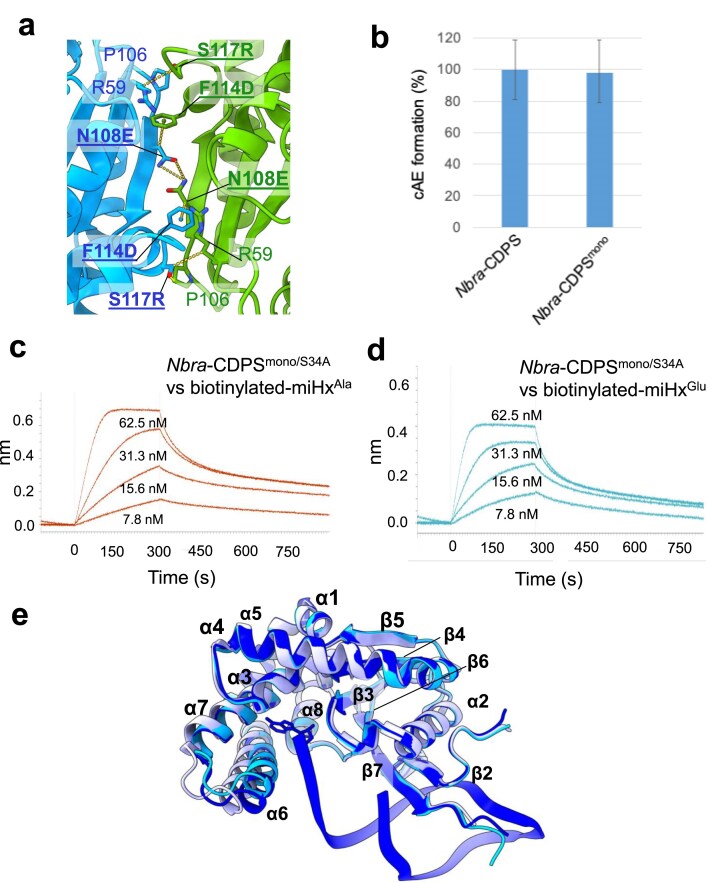
Characterization of *Nbra*-CDPS and its variants. (a) Detailed view of the *Nbra*-CDPS dimerization interface, predominantly mediated by residues N108, F114, and S117 (PDB 5MLQ). A multi-sequence alignment of CDPSs forming cAE revealed that these positions differ between monomeric and dimeric enzymes within this family (SI Fig. 5a-b). We constructed the N108E/F114D/S117R variant, which proved to be monomeric and was named *Nbra*-CDPS^mono^ (SI Fig. 5c). (b) *In vitro* cAE-synthesizing activity of *Nbra*-CDPS and *Nbra*-CDPS^mono^ determined by end-point assays. Experiments were carried out in duplicate. (c-d) Affinity measurements of the interaction using BLI. The monomeric inactive variant *Nbra*-CDPS^mono/S34A^ was tested at four concentrations (7.8, 15.6, 31.3, and 62.5 nM) using biotinylated miHx^Ala^ (c) or miHx^Glu^ (d). Experiments were made in triplicate. (e) Superimposition of the high-resolution untagged apo *Nbra*-CDPS^S34A^ structure obtained in this work (PDB: 9I5M, 1.7 Å; cyan) with the previously reported apo *Nbra*-CDPS structure (PDB: 5MLQ, 3.2 Å; lavender) and the *Nbra*-CDPS/miHx^Ala^ complex (PDB: 9IAM, 3.6 Å; dark blue). The overlay reveals a conformational shift in helix α6.

*Nbra*-CDPS was expressed with or without a C-terminal His-tag. The *Nbra*-CDPS dimeric interface involves residues including N108, F114, and S117 (Fig. 2a and SI Fig. 5a). We performed a multiple sequence alignment of the eight CDPSs previously shown to synthesize cAE [9] (SI Fig. 5b). We noticed that the residues corresponding to *Nbra*-CDPS N108, F114, and S117 are, respectively, E, D, and R in the sequence from *Bulkholderia lata* (*Blat*-CDPS previously called CDPS 37) that is monomeric. We therefore engineered the *Nbra*-CDPS variant carrying N108E, F114D, and S117R mutations. This variant was purified as a soluble monomer, as determined by Size Exclusion Chromatography (SEC) (SI Fig. 5c). Importantly, the monomeric variant (*Nbra*-CDPS^mono^) displayed cyclodipeptide-synthesizing activity comparable to that of the dimeric wild-type enzyme, validating its use for further structural and functional analyses (Fig. 2b). Catalytically inactive variants were also generated by replacing the active site serine residue S34, required for the aminoacyl-enzyme formation, with an alanine. These mutants were called *Nbra*-CDPS^S34A^ and *Nbra*-CDPS^mono/S34A^ (SI Fig. 5d). The protein stability of these variants was assessed by nano-Differential Scanning Fluorometry (nanoDSF) (SI Fig. 5e,f). Both inactive variants were thermally stable, and notably, the monomeric *Nbra*-CDPS^mono/S34A^ exhibited higher thermal stability than the dimeric *Nbra*-CDPS^S34A^, with Inflection Temperatures (Ti) of 50.3°C and 45.5°C, respectively.

### Interactions of *Nbra*-CDPS with miHxs that mimic the acceptor arms of tRNA^Ala^ or tRNA^Glu^

We previously measured the catalytic activity of *Nbra*-CDPS in the presence of Ala-miHx^Ala^ and Glu-miHx^Glu^ and showed that aminoacylated miHxs (AA-miHxs) were as effective a substrate as entire AA-tRNAs [9]. We next aimed to further characterize their molecular interactions. The miHx^Ala^ presents an A^73^CCA^76^ 3′ tail, a helical stem (formed by 5′-G^1^GGGCUA^7^ that hybridizes with the sequence 5′-U^66^AGCUCC^72^ (tRNA^Ala^ numbering) and a loop (noted 5′-U^8^UCG^11^ hereafter), known to enhance the stability of miHxs (Fig. 1c). The miHx^Ala^ presents a wobble between bases G^3^ and U^70^, a hallmark of the tRNA^Ala^ acceptor stem [29]. The miHx^Glu^ has the same elements, but with different sequences, and in particular a canonical C^3^-G^70^ Watson-Crick pair (Fig. 1c). We synthesized the aminoacylated forms, Ala-miHx^Ala^ and Glu-miHx^Glu^, using flexizymes (Fxs). The AA-miHxs were then purified on DNAPAC columns to reach 70% or 80% of the aminoacylated forms of miHx^Ala^ or miHx^Glu^, respectively. The acylation reaction is reversible due to the spontaneous hydrolysis of the ester bond between the amino acid and the miHx. The half-life of Ala-miHx^Ala^ and Glu-miHx^Glu^, i.e. their spontaneous hydrolysis in miHx^Ala^ and miHx^Glu^, was previously measured to be 60 and 145 min, respectively, at pH 7.5 and 20°C [9]. To circumvent this instability, we also generated Ala-(amide)-miHx^Ala^ in which the ester bond is replaced by a stable amide linkage, following a previously described protocol [24]. In this approach, the terminal nucleotide A^76^ bears an amine group instead of the 3′-OH, allowing reaction with Fx to load Ala onto miHx and form a stable amide bond with the ribose (SI Fig. 2b,c). In the case of Ala-(amide)-miHx^Ala^, we did not observe any degradation over time, including at room temperature, thus confirming its high stability.

To probe the interactions between *Nbra*-CDPS^mono/S34A^ and miHx^Ala^, we first performed electrophoretic mobility shift assays (EMSA) using miHx^Ala^ labelled with 6-FAM (6-carboxyfluorescein) on position 10 in the 5′-U^8^UCG^11^ loop. We observed an interaction in the nanomolar range. The miHx was fully saturated with protein at concentrations larger than 300 nM (SI Fig. 6). To measure the interaction with an orthogonal approach quantitatively, we used BioLayer Interferometry (BLI). We used a miHx^Ala^ containing a biotin group at the same position 10 in the 5′-U^8^UCG^11^ loop and loaded on BLI tips coated with streptavidin. We measured a nanomolar affinity with a K_D_ value of 14.6 ± 4.2 nM between *Nbra*-CDPS^mono/S34A^ and the miHx^Ala^ (Fig. 2c). The same experiment, but using miHx^Glu^ instead of miHx^Ala^, gave a *K*_D_ value of 12 ± 2 nM, showing very similar affinities for the two miHxs (Fig. 2d).

### Crystal structure of *Nbra*-CDPS bound to a miHx^Ala^

**Figure 3.**
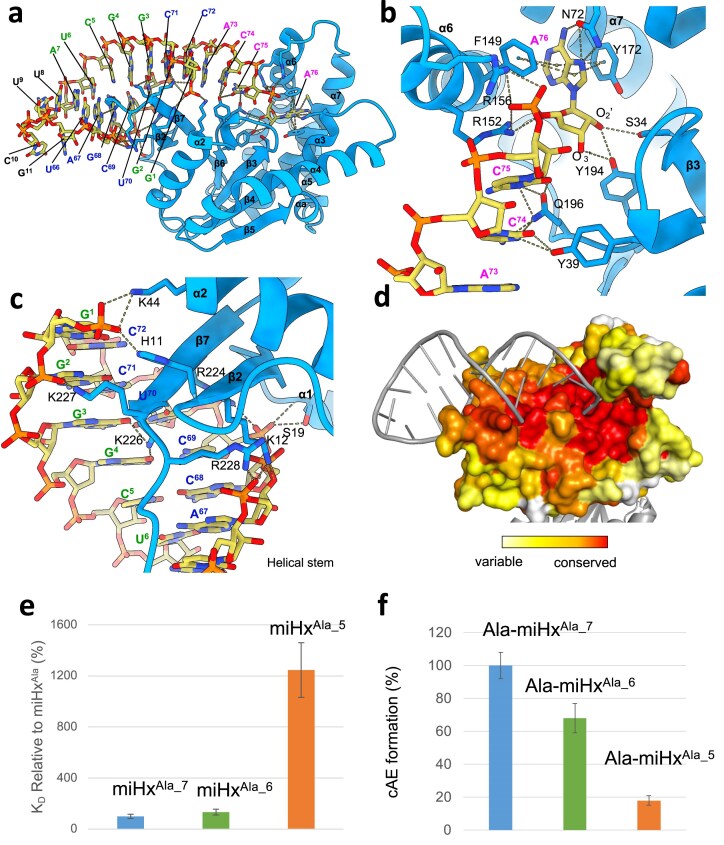
Characterization of the interaction between *Nbra*-CDPS and miHx^Ala^. (a) Overall view of the crystal structure of the *Nbra*-CDPS/miHx^Ala^ complex, colored, respectively, in cyan and yellow. Nucleotides are numbered as in Fig 1c; nucleotide labels are colored magenta for the 3′ ACCA tail, green for the 5′ strand, blue for the complementary 3′ strand, and black for the loop connecting the two strands. (b) Close-up of the interaction site of the 3′ ACCA tail. (c) Close-up of the interaction site of the 7 bp helical stem of miHx^Ala^. (d) Surface representation showing amino acid conservation in the XYP CDPS family; residues are colored on a gradient from red (conserved) to white (non-conserved). (e) Dissociation constants K_D_ of *Nbra*-CDPS^mono/S34A^ with miHx^Ala^ of different stem lengths (7 bp (miHx^Ala_7^), 6 bp, and 5 bp) measured by BLI. K_D_ values are normalized to miHx^Ala_7^ (set at 100%). (f) *In vitro* cAE-synthesizing activity of *Nbra*-CDPS with shortened Ala-miHx^Ala^, reported as percentages relative to miHx^Ala_7^ (set as 100%). Experiments were carried out in duplicate.

We first determined the structure of an apo form of *Nbra*-CDPS^S34A^. This crystal diffracted to 2.8 Å and the refined structure is very similar to that previously reported at 3.2 Å (PDB 5MLQ) [17]. We optimized crystallization conditions and determined the crystal structure with an untagged apo form of *Nbra*-CDPS^S34A^ that diffracted at 1.7 Å (SI Table 2). This structure aligns closely with the previous one (PDB 5MLQ) [17], with an overall RMSD of 0.6 Å over 210 Cα atoms. The most notable conformational change is observed in the orientation of helix α6 (Fig. 2e), illustrating some structural plasticity for this helix. We also determined the structure of *Nbra*-CDPS bound to miHx^Ala^ at 3.3 Å resolution (Fig. 3a, SI Table 2). In the complex, the 3′ tail of miHx^Ala^ interacts with catalytic residues S34, Y172, and Y194 (Fig. 3b), corresponding to S37, Y178, and Y202 in *Snou*-CDPS (SI Fig. 1), at the upper region of the P1 pocket. The 3′-CCA tail deviates from the acceptor stem axis (Fig. 3a). A^76^ is stabilized by π-stacking with F149 and Y172, while Y194 forms hydrogen bonds with its O2′ and O3′ atoms, and S34 with its O2′. The A^76^ base also forms hydrogen bonds with N72 and C169.

The second main patch of interaction is mediated by the acceptor stem part of the miHx^Ala^ formed by the helix between ^5′^G^1^GGGCU^6^ and ^5′^A^66^GCUCC^72^, preceding the 3′-CCA tail (Fig. 3c). Residues from the *N*-terminal strand β_2_ and the *C*-terminal strand β_7_ of *Nbra*-CDPS interact with the major groove of the RNA helix. Salt bridges are observed between *Nbra*-CDPS residues H11, K12, S19, K44, R224, K227, and R228 and the phosphate groups of nucleotides G^1^, A^67^, G^68^, C^69^, and U^70^. Bases G^4^ and A^67^ further establish hydrogen bonds with K226 and K229, respectively. To assess whether this intermolecular interface is preserved in solution, we probed binding of *Nbra*-CDPS^mono/S34A^ to miHx^Ala^ by NMR spectroscopy. Despite the limited chemical shift assignment range available for the protein, we could demonstrate that the regions of the CDPS involved in miHx^Ala^ binding are conserved in solution (SI Fig. 7 for buffer optimization for NMR, SI Fig. 8 for NMR data, and the experimental procedures are provided in the Supp Methods).

Electrostatic surface representation of *Nbra*-CDPS shows that the stem and the 3′-tail of miHx^Ala^ interact predominantly with positively charged residues of the enzyme (SI Fig. 9a). Mapping evolutionary conservation across XYP family CDPSs producing cAE revealed that the miHx^Ala^-interacting surface is enriched in highly conserved residues (Fig. 3d). Structures of apo and miHx^Ala^ bound structures superimpose well (RMSD of 0.44 Å over 212 Cα atoms). The main conformational change is on helix α6 (amino acids F149 to S155). This helix moves away from the active site in the presence of miHx^Ala^ (SI Fig. 9b), confirming its high structural adaptation to the local conditions. In this region, R152 and R156 establish salt bridges with the phosphate groups of A^76^, while R152 additionally engages in π–cation interaction with C^75^. R152 forms hydrogen bonds with Q196 in the apo state, which are disrupted in the complex, enabling recognition of the 3′-ACCA tail. Finally, conformational rearrangement of Y39 allows stacking interactions with C^74^ and C^75^ pyrimidine rings.

### Minimal helical region of miHx^Ala^ required for *Nbra*-CDPS interaction and catalysis

In the *Nbra*-CDPS/miHx^Ala^ crystal structure, the enzyme contacts bases G^1^, G^3^, and G^4^ on one strand and A^67^, G^68^, C^69^, and U^70^ on the opposite strand (Fig. 3c). To define the minimal size of the helical region required for binding, we measured by BLI the affinity of *Nbra*-CDPS^mono/S34A^ for progressively shortened miHx^Ala^ constructs. Deletion of the terminal A^7^-U^66^ base pair (miHx^Ala_6^) did not significantly affect binding, with an affinity close to that of the full-length (K_D_ of 18 ± 6 nM for miHx^Ala_6^ versus K_D_ value of 14.6 ± 4.2 nM for miHx^Ala_7^) (Fig. 3e). This observation is consistent with the absence of direct contacts between the terminal base pair of miHx^Ala^ and *Nbra*-CDPS. Further truncation, removing the U^6^-A^67^ base pair (miHx^Ala_5^), resulted in a weaker binding with a ∼10-fold increase in K_D_ (K_D_ of 171 ± 58 nM) (Fig. 3e). This decrease in affinity correlates with the loss of contacts between the U^6^-A^67^ base pair and *Nbra*-CDPS residues K12, R228, and K229, as suggested by the crystal structure.

We next assessed the catalytic activity of *Nbra*-CDPS on aminoacylated truncated miHxs constructs. We observed a slight decrease in activity with Ala-miHx^Ala_6^ and a strong decrease with Ala-miHx^Ala_5^ (Fig. 3f). In a previous study, we measured a 10-fold decrease in catalytic activity observed for miHx^Ala_4^ compared to the reference miHx^Ala^, which corresponds to a two-fold decrease in activity compared to miHx^Ala_5^. The activity of *Nbra*-CDPS was also measured on an Ala-RNA^(5′-ACCA)^ corresponding to the 3′-tail of the arm without stem, and no catalytic activity was detected [9]. We thus showed that the stem of the CDPS substrate is essential for activity, as it mediates multiple intermolecular interactions that stabilize complex formation, whereas the 3′-ACCA tail is required but likely not sufficient to ensure proper positioning of the aminoacyl group for activity.

### Crystal structure of *Nbra-*CDPS with Ala-(amide)-miHx^Ala^ and modelling of the alanylated-enzyme

**Figure 4.**
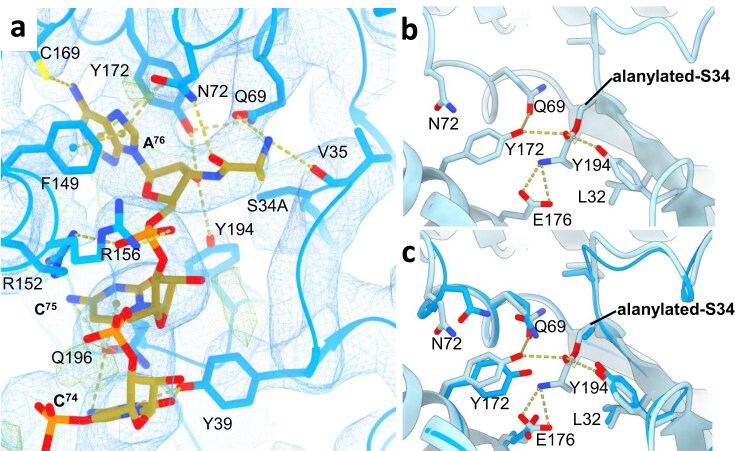
Structural insights into the *Nbra*-CDPS^S34A^/Ala-(amide)-miHx^Ala^ complex and the alanyl-enzyme intermediate. (a) Electron density map of the 3′-ACCA tail of Ala-(amide)-miHx^Ala^ in complex with *Nbra*-CDPS^S34A^. The 2Fo-Fc map is in blue (1.5σ) and the Fo-Fc map in green (positive) and red (negative) at 3σ. (b) Active-site view of the catalytic intermediate featuring the alanylated enzyme at residue S34, obtained from molecular simulation. The distance distribution between the *N*-terminal atom of the alanine moiety and the carboxylate group of the residue E176 and between the phenol oxygen of Y172 and the oxygen of the C = O group of the ester bond is provided in the SI Fig. 11b–f. (c) Superimposition of the *Nbra*-CDPS active site observed in the *Nbra*-CDPS^S34A^/Ala-(amide)-miHx^Ala^ complex with that of the alanyl-enzyme intermediate obtained from molecular simulation.

We determined the crystal structure at 3.6 Å of *Nbra*-CDPS^S34A^ in complex with Ala-(amide)-miHx^Ala^, in which a stable covalent bond is formed between the Ala and miHx^Ala^ moieties (SI Fig. 2c). The crystals display the same unit cell parameters as those obtained for the *Nbra*-CDPS^S34A^/miHx^Ala^ complex (Table S2). The electron density map revealed an alanine covalently bound to the N^3’^ of miHx^Ala^ (Fig. 4a). In this structure, the terminal nucleotide A^76^ adopts the same position as in the *Nbra*-CDPS/miHx^Ala^ complex, stacked between residues Y172 and F149 (Fig. 4a). The carbonyl group of the alanine moiety forms a hydrogen bond with residue N72 of *Nbra*-CDPS. The amine group of the alanine moiety establishes hydrogen bonds with residues V35 and Q69, the latter also engaging in hydrogen bonding with the catalytic residue Y172. Additionally, the alanine methyl group is positioned near the carbonyl oxygen and Cα atoms of V35 and G36. Notably, Q69 and N72, together with V35 and G36, belong to the two distinct and highly conserved surface patches characteristic of cAE-synthesizing CDPS enzymes. To further confirm the covalent attachment of alanine to miHx^Ala^, we calculated a Fo-Fc omit map, which clearly shows the electron density corresponding to the CCA-Ala moiety (SI Fig. 10). This map, contoured at 3.0σ, confirms both the presence and the orientation of the alanine residue, consistent with the hydrogen bonding network involving Q69 and N72.

We also performed a molecular modelling study of the catalytic intermediate, in which the enzyme is covalently alanylated on residue S34 *via* an ester bond (Fig. 4b, SI Figs 11 & 12a). The initial model was refined by molecular dynamics. According to the proposed mechanism, two interactions are important: (i) the *N*-terminal atom of the acyl-alanine moiety (N2) (SEAO1) with the carboxylate group of the residue E176 (E176OE1) and (ii) the phenol oxygen of Y172 (Y172OH) in interaction with the oxygen of the C = O group of the ester bond (O1) (SEA34O1). The distribution of these distances in the 0.2 µs Ala-*Nbra*-CDPS MD simulation is shown in the SI Fig. 12b–d. In this trajectory, a fraction of the extracted conformers displays distances between d[SEA34O1_Y172OH] and d[SEA34N1_E176OE1/OE2] comprised respectively between 2.3 and 3.5 Å. This is consistent with an interaction (hydrogen bond or salt bridge) between the corresponding chemical groups. Monitoring the distance between the methyl group of the acyl-Ala of SEA34 and residue L32 in the P1 pocket clearly shows that, in this intermediate state, the methyl group of acyl-Ala of SEA34 occupies the P1 pocket (SI Fig. 12e,f). The model of the acylated enzyme superposes well with the crystal structure of the enzyme bound to the Ala-(amide)-miHx^Ala^. Hence, acylation does not induce any significant conformational change in the CDPS active site (Fig. 4c). The Ala bound to S34 is positioned as expected in the P1 pocket. Acylation of S34 leads to a reorientation of the E176 OH in the acyl-intermediate, stabilizing the NH of alanyl bound to the S34 in the active site.

### The cryo-EM structure of *Nbra*-CDPS^S34A^ with tRNA^Ala^

**Figure 5.**
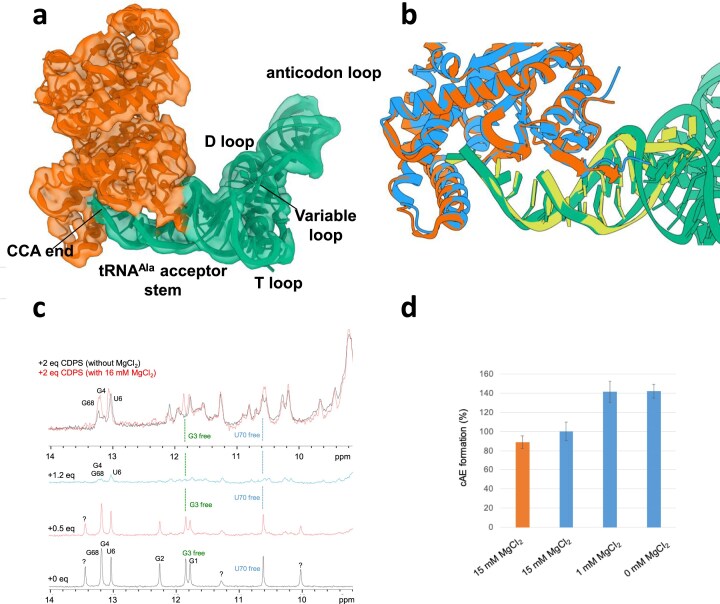
Structural comparison of tRNA^Ala^ and miHx^Ala^ binding to the enzyme and impact of MgCl₂ concentration on complex formation and catalytic activity. (a) Single-particle cryo-EM map with the model of *Nbra*-CDPS^S34A^/tRNA^Ala^ fitted in the map. The tRNA^Ala^ is represented in green, the *Nbra*-CDPS^S34A^ dimer in orange. (b) Superimposition of the tRNA acceptor stem with the miHx^Ala^ in complex with *Nbra*-CDPS^S34A^. The tRNA^Ala^ and miHx^Ala^ are colored, respectively, in green and yellow. The *Nbra*-CDPS^S34A^ and *Nbra*-CDPS bound, respectively, to the tRNA and miHx^Ala^ are colored, respectively, in orange and blue. (c) 1D ^1^H spectra of miHx^Ala^ collected in the presence of increasing concentrations of *Nbra*-CDPS^mono/S34A^ at 0 mM MgCl_2_ concentration. In the presence of an increasing concentration of *Nbra*-CDPS^mono/S34A^, the imino signals from free miHx^Ala^ decrease in overall intensity in agreement with the formation of a complex (see for comparison the spectra recorded in the presence of 0, 0.5, and 1.2 molar equivalents of *Nbra*-CDPS^mono/S34A^). When we added 16 mM concentration of MgCl₂, the signals corresponding to the free miHx^Ala^ reappeared (see G^3^ free and U^70^ free, for example), suggesting a weakened interaction. This is evident in the top spectrum, which is a superposition of the spectra recorded in the presence of 2 molar equivalents of *Nbra*-CDPS^mono/S34A^, in the absence (in black) and in the presence of 16 mM MgCl₂ (in red). The spectra were recorded with 256 scans (NS) in the presence of 0, 0.5, and 1.2 molar equivalents of *Nbra*-CDPS^mono/S34A^, and with 2048 scans in the presence of 2 molar equivalents to increase the signal-to-noise ratio. (d) Influence of MgCl_2_ concentration on cAE-synthesizing activity of *Nbra*-CDPS. End-point assays were performed under standard conditions using Ala-miHx^Ala^ and Glu-tRNA^Glu^ in the presence of 15 mM MgCl_2_ (orange). The same assays were performed using Ala-tRNA^Ala^ instead of Ala-miHx^Ala^ in the presence of 15, 1, and 0 mM MgCl_2_ (blue). Experiments were carried out in duplicate.

To further assess how miHx^Ala^ structurally relates to the natural substrate, we determined the cryo-EM structure of *Nbra*-CDPS in complex with a full-length tRNA^Ala^. For this purpose, we assembled a complex of dimeric *Nbra*-CDPS^S34A^ and tRNA^Ala^, first optimizing conditions by negative staining. Cryo-EM grids were then prepared using a molar ratio of 1:2.7 (*Nbra*-CDPS^S34A^:tRNA^Ala^). Image analysis yielded 2D classes of particles, corresponding to a dimer of *Nbra*-CDPS^S34A^ bound to a single tRNA^Ala^ molecule. Local refinement of the selected particles obtained after heterogeneous refinement produced a cryo-EM map at 4.2 Å resolution, into which a dimer of *Nbra*-CDPS^S34A^ and one tRNA^Ala^ were fitted (Fig. 5a) (SI Fig. 13). Superimposition of the crystal structure of the *Nbra*-CDPS^S34A^/miHx^Ala^ complex onto the cryo-EM structure of *Nbra*-CDPS/tRNA^Ala^ revealed that the miHx overlays precisely with the acceptor stem of the full tRNA^Ala^ (Fig. 5b). This finding demonstrates that the miHx is a faithful structural mimic of the tRNA acceptor stem within the CDPS family.

### Impact of magnesium ions on *Nbra*-CDPS/miHx^Ala^ interaction and catalytic activity

Mg^2+^ is routinely added to enzymatic and binding assays involving tRNA, including CDPS systems, as they usually stabilize tRNA structure [28]. To evaluate the effect of Mg^2+^ on substrate recognition, we measured the affinity of *Nbra*-CDPS for miHx^Ala^ by biolayer interferometry (BLI) while varying MgCl_2_ concentrations from 0 to 4 mM. The affinity decreases linearly as MgCl_2_ concentration increases (*K*_D_ of 14.6 ± 4.2 nM without MgCl_2_ to 950 ± 100 nM at 4 mM), indicating that high Mg^2+^ concentrations weaken the interaction between *Nbra*-CDPS and miHx^Ala^ (SI Fig. 14a). This result was confirmed by NMR. Indeed, after the formation of the complex at 0 mM MgCl_2_ concentration, the addition of 16 mM MgCl_2_ led to the reappearance of free miHx^Ala^ (Fig. 5c). NMR further showed that the structure of miHx^Ala^ is not significantly modified upon MgCl_2_ addition, excluding RNA unfolding as a dominant factor for the MgCl_2_-induced complex weakening (SI Fig. 14b,c). NMR chemical shift perturbation further reveals preferential Mg^2+^ binding at the 5′-end of miHx^Ala^ (SI Fig. 14b,c). To further assess the impact on enzyme activity, we measured the cAE formation catalysed by *Nbra*-CDPS in the presence of both substrates at increasing concentrations of MgCl_2_ (Fig. 5d). We observed optimal cAE production in the absence of MgCl₂ or at 1 mM MgCl₂, which is not surprising since the tRNAs used were already properly folded and enzymatic activity does not require MgCl_2_. However, activity was reduced by 40% in the presence of 15 mM MgCl₂. Taken together, our data support a millimolar-range binding of Mg^2+^ cations at the 5′-end of miHx^Ala^, which in turn weakens its interaction with *Nbra*-CDPS and reduces cyclodipeptide-synthesizing activity.

### Convergent binding modes of miHx^Glu^ and miHx^Ala^ on *Nbra*-CDPS

**Figure 6.**
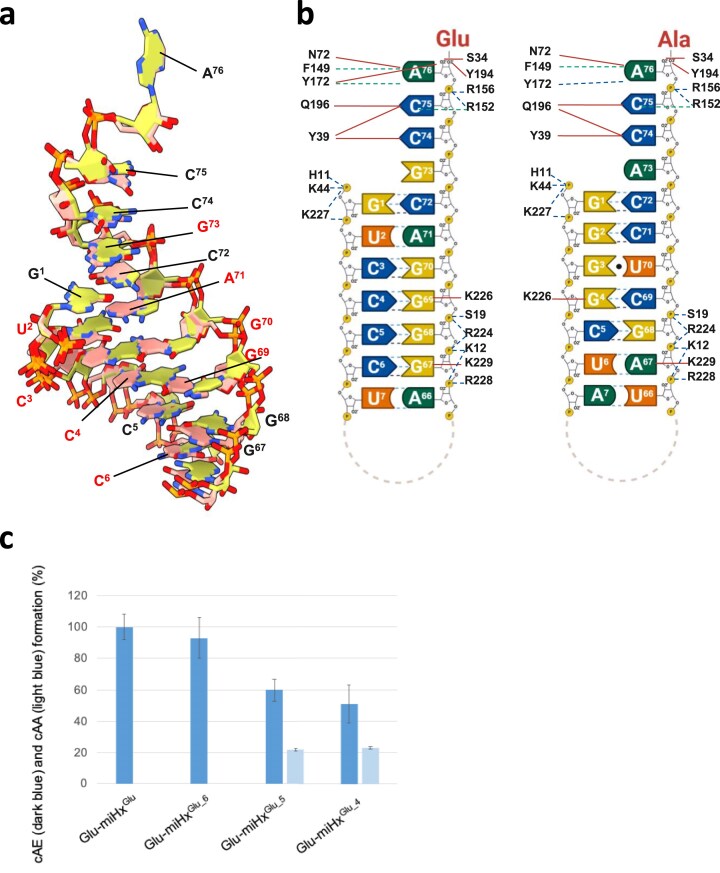
Characterization of the interaction between *Nbra*-CDPS and miHx^Glu^, and comparison with its interaction with miHx^Ala^. (a) Superimposition of miHx^Glu^ (salmon) and miHx^Ala^ (yellow) structures in complex with *Nbra-*CDPS (sequence shown is the one from miHx^Glu^). Nucleotides shown in red indicate sequence differences between the two miHxs. (b) Secondary structures of miHx^Glu^ and miHx^Ala^ and their respective contacts with *Nbra*-CDPS. Interaction analyses were performed using PLIP, PISA (implemented in CCP4), and Nucplot. The 2D schematic of the interactions was created in BioRender, Marouf, Z. (2026) https://BioRender.com/o7nus1k. Hydrogen bonds are depicted as solid red lines, salt bridges as blue dashed lines, and π–π or π–cation interactions as green dashed lines. (c) Cyclodipeptide-synthesizing activity of *Nbra*-CDPS in the presence of shortened Glu-miHx^Glu^. End-point assays were performed under standard conditions in duplicate. In addition to cAE production (dark blue), cAA production (light blue) is also detected when Glu-miHx^Glu_5^ and Glu-miHx^Ala_4^ are used.

We determined the crystal structure of *Nbra*-CDPS in complex with miHx^Glu^. We obtained two crystal forms that diffracted at 3.4 and 3.5 Å resolution under conditions similar to those used for the *Nbra*-CDPS/miHx^Ala^ complex (Supp Methods). The overall fold of the *Nbra*-CDPS/miHx^Glu^ complex closely resembles that of *Nbra*-CDPS bound to miHx^Ala^, with an RMSD of 0.35 Å over 226 Cα atoms. A comparable conformational change of helix α6 was observed upon miHx^Glu^ binding as with miHx^Ala^. Superimposition of the two complexes revealed that miHx^Glu^ and miHx^Ala^ adopt nearly identical binding modes despite their sequence differences (Fig. 6a, see also Fig. 1c). Remarkably, the bent 3'-XCCA tail, including nucleotide A^76^ adopts the same position and contacts *Nbra*-CDPS similarly in both complexes, despite the flexibility of this region detected by NMR (Fig. 6b).

The high degree of superimposition was unexpected, given the presence of a wobble pair (G^3^-U^70^) in miHx^Ala^ that is absent in miHx^Glu^. Notably, the O6 and N7 atoms of G^69^, located in the fourth base pair of miHx^Glu^, align with those of G^4^ in miHx^Ala^ on the opposite strand, thereby maintaining the interaction with K226. A comparable cross-strand alignment of acceptor atoms was observed at base pair C^3^-G^70^. Furthermore, K229 forms a conserved hydrogen-bond interaction with nucleotide 67 in both miHx^Ala^ (A^67^) and miHx^Glu^ (G^67^), indicating that both miHxs adapt to preserve this conserved interaction. Beyond these subtle differences, most contacts between the miHxs and *Nbra*-CDPS are conserved, reflecting the importance of phosphate backbone interactions (Fig. 6b).

We also assessed the catalytic activity using Glu-miHx^Glu^ mutants of different helical lengths (Fig. 6c). Reducing the helical stem length progressively decreases activity, although the effects were less pronounced than with the Ala-miHx^Ala^ mutants. Specifically, the enzyme retains ∼ 60% of its activity with Glu-miHx^Glu_5^ (two base-pairs removed), whereas Ala-miHx^Ala_5^ supported only ∼ 20% of activity. Some cAA cyclodipeptides are produced when the size of the Glu-miHx^Glu^ is reduced, suggesting that Ala-miHx^Ala^ may compete more effectively as a second substrate under these conditions. This observation is consistent with a shift in substrate usage likely influenced by differences in affinity, although further kinetic analyses would be needed to confirm the underlying mechanism.

### Identification of key recognition determinants on *Nbra*-CDPS substrates

**Figure 7.**
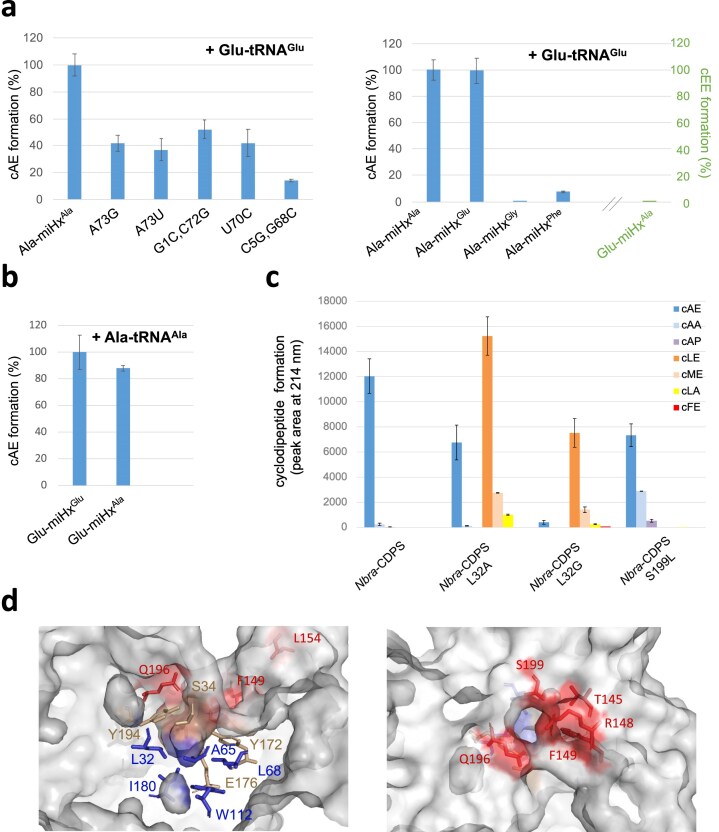
Specific recognition between *Nbra*-CDPS and its two substrates. (a) *In vitro* cyclodipeptide synthesis by *Nbra*-CDPS in the presence of alanylated mutated miHx^Ala^ (left) and misacylated miHxs (right). End-point assays were performed under standard conditions using Glu-tRNA^Glu^ as the other substrate. It is worth noting that the misacylated Glu-miHx^Ala^ was also tested in the presence of Glu-tRNA^Glu^, but no cyclodipeptide—particularly cEE—was detected (shown in green). (b) *In vitro* cyclodipeptide synthesis by *Nbra*-CDPS using misacylated Glu-miHx^Ala^ and Ala-tRNA^Ala^ as substrates under standard conditions was carried out in duplicate. (c) *In vivo* cyclodipeptide production by *Nbra*-CDPS and selected variants. Assays were performed *in vivo* to detect all cyclodipeptides produced and were carried out in triplicate. Note that no cGE was detected. (d) Close-up view of the P1 pocket, composed of five residues (left) and the C2 pocket, also composed of five residues (right) in *Nbra*-CDPS (crystal structure obtained in this work, 9I5M) (see also SI Fig. 1c prepared using the previously reported crystal structure, 5MLQ).

We measured the affinity and catalytic activities of *Nbra-*CDPS with different miHx^Ala^ mutants (Fig. 7a left and SI Fig. 15a). When the Ala-miHx^Ala/A73G^ mutant (in which A^73^ is replaced with G, as in miHx^Glu^) was used instead of Ala-miHx^Ala^, *Nbra*-CDPS retained 80% of its affinity and 40% of its activity. A similar effect was observed with Ala-miHx^Ala/A73U^. We next tested the double mutant Ala-miHx^Ala/G1C-C72G^, in which the first base pair of the acceptor stem is modified, a position previously reported to be essential for another CDPS [30]. *Nbra*-CDPS maintained more than 80% binding affinity and ∼50% catalytic activity on this substrate, indicating a more relaxed requirement for this base pair in *Nbra*-CDPS.

Finally, we used the Ala-miHx^Ala/U70C^ to investigate the contribution of the G^3^•U^70^ wobble pair, a hallmark of tRNA^Ala^ identity elements recognized by AlaRS [29]. *Nbra*-CDPS retained 16% and 40% of its affinity and activity, respectively, on this substrate, indicating that this mismatch contributes to stabilizing the enzyme/product complex but is not essential for the CDPS specificity. The similar binding affinity of miHx^Ala^ and miHx^Glu^ (K_D_ ∼ 10 nM), but the reduced affinity of Ala-miHx^Ala/U70C^ (K_D_ ∼ 90 nM) to *Nbra*-CDPS suggests that the absence of the wobble in miHx^Glu^ is likely compensated by other sequence elements to maintain high affinity. Together, these data highlight that *Nbra*-CDPS substrate recognition is primarily dictated by sequence elements within the tRNA acceptor arm, with certain identity elements such as the G^3^•U^70^ wobble playing a modulatory but not exclusive role in ensuring efficient binding and catalysis.

### Dissection of *Nbra*-CDPS specificity using misacylated miHxs synthesized with flexizymes

To decouple the recognition of the aminoacyl and tRNA moieties by *Nbra*-CDPS, we exploited the versatility of Fxs, which can catalyse misacylations [31]. We first performed the alanylation of miHx^Glu^, called Ala-miHx^Glu^, and showed that it is as good a substrate as Ala-miHx^Ala^, when used together with Glu-tRNA^Glu^. This result is consistent with the similar affinities observed for miHx^Ala^ and miHx^Glu^ (Fig. 7a, right and SI Fig 15b). Conversely, we used the misacylated Glu-miHx^Ala^ in the presence of Ala-tRNA^Ala^ and observed comparable cAE formation (Fig. 7b). This clearly shows that the RNA moieties of both *Nbra*-CDPS substrates can be interchanged without affecting activity, even though only two base pairs (G^1^-C^72^ and C^5^-G^68^) are common between the two miHx sequences. This suggests also that the two substrates are discriminated based on their aminoacyl moieties. As *Nbra*-CDPS does not synthesize cEE, Glu-tRNA^Glu^ cannot be used as either the first or second substrate. It was previously suggested that it cannot act as the first substrate, notably because the glutamyl moiety does not fit into the P1 pocket, which is too small and hydrophobic [9, 11] (SI. Fig. 1). To confirm this, we used Glu-miHx^Ala^ in the presence of Glu-tRNA^Glu^ and observed no cEE formation (Fig. 7a, right, in green), indicating that the glutamyl moiety prevents its use as the first substrate. We then reasoned that the small P1 pocket should be able to accommodate a glycyl moiety (Fig. 7d, left and SI Fig. 1). However, the enzyme does not synthesize cGE (Fig. 7c). We therefore alanylated miHx^Gly^ but the resulting Ala-miHx^Gly^ mutant is not used at all by *Nbra*-CDPS, clearly demonstrating that the miHx^Gly^ sequence is not recognized (Fig. 7a, right).

To assess the impact of the P1 pocket size and composition on the recognition of the first substrate, we engineered *Nbra*-CDPS variants with an expanded P1 pocket (see Fig. 7d, left and SI Fig. 1). We constructed the *Nbra*-CDPS^L32A^ and *Nbra*-CDPS^L32G^ variants in which leucine 32 is replaced by less bulky alanine or glycine residues. As expected, these variants accommodate a leucyl moiety in P1, as shown by the appearance of cLE synthesis at the expense of cAE synthesis (Fig. 7c). Interestingly, these variants synthesize little or no cFE, whereas many cLE-synthesizing CDPSs also synthesize cFE [13]. We then alanylated miHx^Phe^ and showed that *Nbra*-CDPS produces only 8% cAE when it uses this mutant in place of the Ala-miHx^Ala^ (Fig. 7a, right). This is also in agreement with the interaction measurement that showed a weakened interaction for miHx^Phe^ (SI Fig. 15b). Comparison of the miHx^Gly^ and miHx^Phe^ sequences reveals differences at positions 3 and 4, but both share base pairs G^1^-C^72^, C^2^-G^71^, and G^5^-C^68^ (Fig. 1c). Since G^1^-C^72^ is present in both miHx^Ala^ and miHx^Glu^, and C^2^-G^71^ is likely permissive because U^2^-A^71^ from miHx^Glu^ is tolerated, the G^5^-C^68^ base pair may be a key determinant. We therefore alanylated the miHx^Ala/C5G,G68C^ mutant and observed that *Nbra*-CDPS retained only 14% of its activity with this substrate (Fig. 7a, right), indicating that this base pair is largely responsible for the loss of activity observed when Ala-miHx^Gly^ and Ala-miHx^Phe^ were used. We also modified the C2 cleft and constructed the *Nbra*-CDPS^S199L^ variant in which serine 199 is replaced by a leucine to make the C2 cleft more hydrophobic (Fig. 7d, right). This favors the interaction of the alanyl moiety, as the variant synthesizes more cAA at the expense of cAE (Fig. 7c). Altogether, these data clearly indicate that the *Nbra*-CDPS specificity is governed by both the aminoacyl and tRNA moieties of its two substrates, with specificity determinants present in their acceptor arms.

## Discussion

Our study defines the molecular determinants governing the interaction of a CDPS with RNA miHxs that mimic the acceptor stems of its two sequential tRNA substrates. By combining X-ray crystallography, NMR, cryo-EM, molecular modelling, and molecular biology, we delineated the two binding regions of the XYP *Nbra*-CDPS, which first accommodate Ala-tRNA^Ala^ and then Glu-tRNA^Glu^. Using miHxs that were mutated, non-acylated, acylated, or misacylated, we identified key substrate determinants that are essential for CDPS recognition.

Our work provides detailed insights into the recognition of the first substrate by XYP CDPSs. Until now, the only structural data available were from the complex between *Cglo*-CDPS and its first substrate Phe-tRNA^Phe^ [22]. This 5 Å structure showed that the acceptor arm stem interacts with the positively charged β2 and β7 strands of the enzyme, while the 3′ tail was not resolved and had only been modelled to fit with the phenylalanyl moiety in the P1 pocket. In our study, we achieved higher-resolution structural information (3.6 Å) on the interaction of Ala-(amide)-miHx^Ala^ with *Nbra*-CDPS. Our structure reveals that the bent orientation of the 3′ tail is fully consistent with the positioning of alanyl in the P1 pocket, in agreement with the molecular dynamics simulation results obtained with the Ala-(ester)-miHx^Ala^ bound to a *Nbra*-CDPS. In addition, our structure precisely identifies the contacts made by the stem with the enzyme, notably with the positively charged residues in the β2 and β7 strands.

We also obtained the first information on the interaction of CDPSs with their second substrate. We solved the structure of *Nbra*-CDPS in complex with miHx^Glu^. Unexpectedly, miHx^Glu^ superimposes perfectly with miHx^Ala^ in the respective enzyme complexes, with its stem interacting with similar residues and its 3′ tail orientated towards P1. However, when acylated, it cannot act as the first substrate because the glutamyl moiety cannot be accommodated in the P1 pocket. We performed the misacylation of miHx^Glu^ and showed that Ala-miHx^Glu^ is as effective a substrate as Ala-miHx^Ala^. These findings clearly indicate that miHx^Glu^ interacts strongly at the binding site of the first substrate, which could also be in part the binding site of the second substrate. Its stem could interact with β2 and β7 strands, and its 3′ tail should reorient to position glutamyl in the C2 cleft. This hypothesis implies restricted conformational changes around the active site between the free and acylated forms of the enzyme. Such a shared binding site for the stem moieties of both substrates would be consistent with the fact that most CDPSs synthesize homocyclodipeptides from two identical AA-tRNAs.

Taken together, our results indicate that XYP CDPSs share a common binding site for the stems of both substrates, with the 3′ tail orienting either toward P1 or C2 to present the first or second substrate, respectively. Substrate selection is determined both by the stem sequence—for instance, *Nbra*-CDPS specifically recognizes the C^5^-G^68^ base pair—and by the identity of the aminoacyl moiety; notably, *Nbra*-CDPS cannot accommodate a glutamyl group in the P1 pocket. It would be interesting to carry out a similar study on NYH CDPSs since no structural data are available on their interaction with a substrate. However, biochemical data and modelling studies clearly show that the first substrate interaction occurs at the positive α4 helix, which is found in all enzymes of the subfamily [32]. It is likely that this binding site also accommodates the second substrate; this hypothesis has already been put forward and supported by molecular modelling studies.

MiHxs have already proven to be valuable tools for dissecting the molecular determinants of AARSs, offering strong complementarity to analyses performed with full tRNAs [33, 34]. Their rigidity has enabled higher-resolution structural data to be obtained compared to AARS–tRNA complexes, and they have been instrumental in probing AARS specificity by testing a wide range of substrates. More recently, they have also been applied to study the molecular mechanisms of other tRNA-dependent enzymes, such as FemX [35]. In the present study, we demonstrated that miHxs, combined with their versatile acylation using Fxs, constitute a tool of choice for investigating CDPS substrate specificity. This approach paves the way for structure-guided engineering of CDPSs to generate an expanded repertoire of molecules, including those incorporating non-canonical amino acids. Interestingly, the same strategy could also be applied more broadly to other tRNA-dependent enzymes.

## Supplementary Material

gkag307_Supplemental_Files

## Data Availability

The crystal structures have been deposited in the Protein Data Bank under the following accession PDB DOIs: untagged *Nbra*-CDPS^S34A^ dimer alone (https://doi.org/10.2210/pdb9i5m/pdb), *Nbra*-CDPS/miHx^Ala^ (https://doi.org/10.2210/pdb9iaj/pdb), *Nbra*-CDPS/miHx^Glu^ form I (https://doi.org/10.2210/pdb9iak/pdb), *Nbra*-CDPS/miHx^Glu^ form II (https://doi.org/10.2210/pdb9ial/pdb), and *Nbra*-CDPS^S34A^/miHx-(amide)-Ala (https://doi.org/10.2210/pdb9iam/pdb).

## References

[1] Canu N, Moutiez M, Belin P. et al. Cyclodipeptide synthases: a promising biotechnological tool for the synthesis of diverse 2,5-diketopiperazines. Nat Prod Rep. 2020;37:312–21. 10.1039/C9NP00036D. 31435633

[2] Gondry M, Sauguet L, Belin P. et al. Cyclodipeptide synthases are a family of tRNA-dependent peptide bond-forming enzymes. Nat Chem Biol. 2009;5:414–20. 10.1038/nchembio.175. 19430487

[3] Moutiez M, Belin P, Gondry M. Aminoacyl-tRNA-utilizing enzymes in natural product biosynthesis. Chem Rev. 2017;117:5578–618. 10.1021/acs.chemrev.6b00523. 28060488

[4] Belin P, Moutiez M, Lautru S. et al. The nonribosomal synthesis of diketopiperazines in tRNA-dependent cyclodipeptide synthase pathways. Nat Prod Rep. 2012;29:961–79. 10.1039/c2np20010d. 22751625

[5] Giessen TW, Marahiel MA. The tRNA-dependent biosynthesis of modified cyclic dipeptides. Int J Mol Sci. 2014;15:14610–31. 10.3390/ijms150814610. 25196600 PMC4159871

[6] Borthwick AD. 2,5-Diketopiperazines: synthesis, reactions, medicinal chemistry, and bioactive natural products. Chem Rev. 2012;112:3641–716. 10.1021/cr200398y. 22575049

[7] Song Z, Hou Y, Yang Q. et al. Structures and biological activities of diketopiperazines from marine organisms: a review. Mar Drugs. 2021;19:403, 10.3390/md19080403 . 34436242 PMC8398661

[8] Jia J, Yao J, Kong J. et al. 2,5-Diketopiperazines: a review of source, synthesis, bioactivity, structure, and MS fragmentation. CMC. 2023;30:1060–85. 10.2174/0929867329666220801143650. 35927899

[9] Canu N, Tellier C, Babin M. et al. Flexizyme-aminoacylated shortened tRNAs demonstrate that only the aminoacylated acceptor arms of the two tRNA substrates are required for cyclodipeptide synthase activity. Nucleic Acids Res. 2020;48:11615–25. 10.1093/nar/gkaa903. 33095883 PMC7672478

[10] Canu N, Belin P, Thai R. et al. Incorporation of non-canonical amino acids into 2,5-diketopiperazines by cyclodipeptide synthases. Angew Chem Int Ed. 2018;57:3118–22. 10.1002/anie.201712536. 29377457

[11] Jacques IB, Moutiez M, Witwinowski J. et al. Analysis of 51 cyclodipeptide synthases reveals the basis for substrate specificity. Nat Chem Biol. 2015;11:721–7. 10.1038/nchembio.1868. 26236937

[12] Yee DA, Niwa K, Perlatti B. et al. Genome mining for unknown-unknown natural products. Nat Chem Biol. 2023;19:633–40. 10.1038/s41589-022-01246-6. 36702957 PMC10159913

[13] Gondry M, Jacques IB, Thai R. et al. A comprehensive overview of the cyclodipeptide synthase Family enriched with the characterization of 32 new enzymes. Front Microbiol. 2018;9:46. 10.3389/fmicb.2018.00046. 29483897 PMC5816076

[14] Schmitt E, Bourgeois G, Gondry M. et al. Cyclization reaction catalyzed by cyclodipeptide synthases relies on a conserved tyrosine residue. Sci Rep. 2018;8:7031. 10.1038/s41598-018-25479-5. 29728603 PMC5935735

[15] Sauguet L, Moutiez M, Li Y. et al. Cyclodipeptide synthases, a family of class-I aminoacyl-tRNA synthetase-like enzymes involved in non-ribosomal peptide synthesis. Nucleic Acids Res. 2011;39:4475–89. 10.1093/nar/gkr027. 21296757 PMC3105412

[16] Moutiez M, Schmitt E, Seguin J. et al. Unravelling the mechanism of non-ribosomal peptide synthesis by cyclodipeptide synthases. Nat Commun. 2014;5:5141. 10.1038/ncomms6141. 25284085

[17] Bourgeois G, Seguin J, Babin M. et al. Structural basis for partition of the cyclodipeptide synthases into two subfamilies. J Struct Biol. 2018;203:17–26. 10.1016/j.jsb.2018.03.001. 29505829

[18] Sutherland E, Harding CJ, Czekster CM. Active site remodelling of a cyclodipeptide synthase redefines substrate scope. Commun Chem. 2022;5:101. 10.1038/s42004-022-00715-2. 36518199 PMC7613923

[19] Harding CJ, Sutherland E, Hanna JG. et al. Correction: bypassing the requirement for aminoacyl-tRNA by a cyclodipeptide synthase enzyme. RSC Chem Biol. 2021;2:942–3. 10.1039/D1CB90009A. 34458818 PMC8341098

[20] Bourgeois G, Seguin J, Babin M. et al. Structural basis for partition of the cyclodipeptide synthases into two subfamilies. J Struct Biol;2018;203:17–26. 10.1016/j.jsb.2018.03.001. 29505829

[21] Aravind L, Anantharaman V, Koonin EV. Monophyly of class I aminoacyl tRNA synthetase, USPA, ETFP, photolyase, and PP-ATPase nucleotide-binding domains: implications for protein evolution in the RNA. Proteins. 2002;48:1–14. 10.1002/prot.10064. 12012333

[22] Bourgeois G, Seguin J, Babin M. et al. Structural basis of the interaction between cyclodipeptide synthases and aminoacylated tRNA substrates. RNA. 2020;26:1589–602. 10.1261/rna.075184.120. 32680846 PMC7566563

[23] Passioura T, Suga H. Flexizymes, their evolutionary history and diverse utilities. Top Curr Chem. 2014;344:331–45. 10.1007/128_2013_421. 23478876

[24] Katoh T, Suga H. Flexizyme-catalyzed synthesis of 3'-aminoacyl-NH-tRNAs. Nucleic Acids Res. 2019;47:e54. 10.1093/nar/gkz143. 30843032 PMC6511858

[25] Peacock JR, Walvoord RR, Chang AY. et al. Amino acid-dependent stability of the acyl linkage in aminoacyl-tRNA. RNA. 2014;20:758–64. 10.1261/rna.044123.113. 24751649 PMC4024630

[26] Murakami H, Ohta A, Ashigai H. et al. A highly flexible tRNA acylation method for non-natural polypeptide synthesis. Nat Methods. 2006;3:357–9. 10.1038/nmeth877. 16628205

[27] Katoh K, Standley DM. MAFFT multiple sequence alignment software version 7: improvements in performance and usability. Mol Biol Evol. 2013;30:772–80. 10.1093/molbev/mst010. 23329690 PMC3603318

[28] Misra VK, Draper DE. The linkage between magnesium binding and RNA folding. J Mol Biol. 2002;317:507–21. 10.1006/jmbi.2002.5422. 11955006

[29] Naganuma M, Sekine S, Chong YE. et al. The selective tRNA aminoacylation mechanism based on a single G•U pair. Nature. 2014;510:507–11. 10.1038/nature13440. 24919148 PMC4323281

[30] Moutiez M, Seguin J, Fonvielle M. et al. Specificity determinants for the two tRNA substrates of the cyclodipeptide synthase AlbC from Streptomyces noursei. Nucleic Acids Res. 2014;42:7247–58. 10.1093/nar/gku348. 24782519 PMC4066775

[31] Goto Y, Katoh T, Suga H. Flexizymes for genetic code reprogramming. Nat Protoc. 2011;6:779–90. 10.1038/nprot.2011.331. 21637198

[32] Croitoru A, Babin M, Myllykallio H. et al. Cyclodipeptide synthases of the NYH subfamily recognize tRNA using an α-helix enriched with positive residues. Biochemistry. 2021;60:64–76. 10.1021/acs.biochem.0c00761. 33331769

[33] Francklyn C, Schimmel P. Aminoacylation of RNA minihelices with alanine. Nature. 1989;337:478–81. 10.1038/337478a0. 2915692

[34] Francklyn C, Shi JP, Schimmel P. Overlapping nucleotide determinants for specific aminoacylation of RNA microhelices. Science. 1992;255:1121–5. 10.1126/science.1546312. 1546312

[35] Fonvielle M, Li de La Sierra-Gallay I, El-Sagheer AH. et al. The structure of FemX(Wv) in complex with a peptidyl-RNA conjugate: mechanism of aminoacyl transfer from ala-tRNA(Ala) to peptidoglycan precursors. Angew Chem Int Ed. 2013;52:7278–81. 10.1002/anie.201301411. 23744707

