# Supplementary material for: The tRNA moieties of both aminoacyl-tRNA substrates of a cyclodipeptide synthase share a common binding site, as revealed by RNA microhelices mimicking tRNA acceptor arms
Source: Nucleic Acids Res. 2026 Apr 7;54(6):gkag307. doi: 10.1093/nar/gkag307 (PMC13062777; doi:10.1093/nar/gkag307)
Supplement: gkag307_Supplemental_Files [file gkag307_supplemental_files.zip › Supplemental Information.pdf]

Supplemental information:

The tRNA moieties of both aminoacyl-tRNA substrates of a cyclodipeptide synthase share a common binding site, as revealed by RNA microhelices mimicking tRNA acceptor arms

Material and methods

Expression and purification of isotopically labeled *Nbra*-CDPS$^{mono/S34A}$ for NMR studies: In order to study the complex *Nbra*-CDPS^{mono}/miHx^{Ala} by NMR, *Nbra*-CDPS$^{mono/S34A}$ was first overexpressed in M9 medium supplemented with $^{15}NH_4Cl$ or both $^{15}NH_4Cl$ and ^{13}C D-Glucose in order to obtain a labelled ^{15}N and ^{15}N-^{13}C protein, essential for NMR studies. The pre-culture was centrifuged (3000 g, 10 min) then resuspended in 10 mL of M9 medium supplemented with $^{15}NH_4Cl$ or both $^{15}NH_4Cl$ and ^{13}C -D-Glucose. Oligo-elements and vitamins were also added using Gibco™ milieu minimum essential (MEM) standard. The suspension was then transferred to a larger volume (500 mL) of the same medium and incubated at 37°C under steering at 200 rpm. When the O.D. at 600nm reached 0.6-0.8, protein expression was induced with 0.5 mM IPTG. The culture was then incubated at 20°C under agitation at 200 rpm for minimum 20 h. The next day, the cell pellets were collected by centrifugation at 3500 rpm on a centrifuge Avanti J-30I with a JLA8.1 rotor for 30 min at 4°C, transferred to PBS 1X buffer and centrifuged for 20 min at 5000 rpm at 4°C before freezing at -80°C.

Deuteration was also used to improve the quality of the tridimensional NMR experiments on *Nbra*-CDPS$^{mono/S34A}$. For production of deuterium-enriched *Nbra*-CDPS$^{mono/S34A}$, 5 mL of a preculture in 2xYT was transferred to 100 mL of 2xYT and the culture was grown at 37°C at 200 rpm to reach an O.D. at 600nm of 1.2. The culture was then centrifuged at 3000 rpm on a Beckman Avanti J-20 centrifuge with a JA-10 rotor at 4 °C for 10 min, and the pellet was resuspended in 500 mL unlabeled M9 medium (containing 4 g/L glucose) and grown at 37°C under stirring at 200 rpm until an O.D. at 600 nm of 0.9. This step allowed the adaptation of bacteria to the minimal medium. The culture was centrifuged again at 3000 rpm at 4°C for 10 min, and the pellet was resuspended in 1 L M9 medium and incubated for 30 min at 37°C at an initial O.D. at 600nm of 0.45. This step allowed purging unlabeled metabolites. The culture was centrifuged again at 3000 rpm at 4°C for 10 min. The pellet was resuspended in 1 L of M9 medium containing ^{15}N-NH_4Cl and ^{13}C labeled (non-deuterated) glucose (2.3 g/L) in 100% D_2O (pH 7.3) at an initial O.D. at 600nm of 0.35. Then, the culture was incubated at 37°C and expression was induced with 0.5 mM IPTG when the O.D. at 600nm reached 0.69. The culture was incubated overnight at 20°C under steering at 200 rpm. After 22h, the cells were harvested and the pellets resuspended in 40 mL of PBS buffer (136.8 mM NaCl, 1.46 mM KH_2PO_4, 8.1 mM Na_2HPO_4, 2.68 mM KCl, pH 7.4) and

centrifuged for 20 min at 6000 rpm on a Beckman Avanti J-20 centrifuge with a JA-10 rotor at 4°C to be stored at -80 °C.

Preparation of aminoacylated miHxs and (amide)-miHxs: Fxs were solubilized in buffer (50 mM HEPES-KOH pH 7.5) and then heated at 95°C for 2 min and slowly cooled down at room temperature for 5 min. The solution was incubated at room temperature for 5 min then on ice for 3 min. The reaction to acylate the 3' end of the miHx was initiated by addition of 5 mM of the corresponding DBE-activated amino acid and incubated on ice for 1 h 45 min for Ala-DBE and 3 h for Glu-DBE. The reaction time was extended to one week for (amide)-miHx due to the low kinetic rate of the acylation. Reactions were quenched by sodium acetate pH 5.2, and miHxs were precipitated by ethanol and stored as a pellet. MiHxs were purified onto a DNAPAC-PA100 semi-preparative column (9 mm x 250 mm, 13.5 µm, ThermoFisher Scientific). Separation was performed under non-denaturing conditions with increasing linear gradients at a flow rate of 5 mL/min, buffer A (25 mM ammonium acetate pH 5.2, 0.5% acetonitrile) and buffer B (2.5 M ammonium acetate pH 5.2, 0.5% acetonitrile). Fractions of interest were desalted to 25 mM ammonium acetate pH 5.2 using a HiTrap 26-10 desalting column (Cytiva), lyophilized and stored at -80°C.

Determination of the cyclodipeptide-synthesizing activities of *Nbra*-CDPS and its variants: For the *in vivo* assay, each protein was overexpressed in *E. coli*; at the end of the cultivation, the culture supernatant was analysed for its cyclodipeptide content. Cyclodipeptides were detected, identified and quantified by LC-MS/MS analyses on an Agilent 1100 HPLC coupled via a split system to an Esquire HCT ion-trap mass spectrometer (Bruker Daltonik GmbH) set in positive mode. Samples were loaded onto a Hypercarb column (4.6 × 150 mm, 5 µm, 250 Å, ThermoScientific) and eluted using a 50 min-linear gradient (100% A to 50% B (v:v) with solvent A: 0.1% (v/v) formic acid in H_2O and solvent B: 0.1% (v/v) formic acid in acetonitrile/H_2O (90/10)) developed at a flow rate of 0.6 mL/min. The cyclodipeptide-synthesizing activities of the variants were determined from at least three-independent experiments. The *in vitro* assay was performed in the presence of purified proteins and aminoacylated miHxs and/or tRNAs. Enzymatic end point tests were conducted as previously described (1). Briefly, they were performed in the following solution: 100 mM sodium phosphate buffer pH 7.5, 50 mM KCl, 15 mM $MgCl_2$ and 0.1 mM ß-mercaptoethanol. Substrates were added to the solution preincubated at 20°C and reactions were started by adding 5 nM *Nbra*-CDPS. After incubation for 30 min, aliquots were withdrawn, acidified with 2% trifluoroacetic acid (TFA) and mixed with known concentrations of stable isotope-labelled internal standards, for quantification of the produced cAE and cAA in LC-MS/MS

analyses. The column was a Hypercarb column (2 × 150 mm, 3 µm, 100 Å ACE) and samples were eluted using a linear gradient from 2 to 42% solvent B at 0.2 mL/min over 20 min. As optimized and defined previously, the substrate concentrations in the assays were 600 nM for Ala-miHx^{Ala} and its mutants in the presence of 600 nM Glu-tRNA^{Glu} or 600 nM for Glu-miHx^{Glu} and its mutants in the presence of 1200 nM Ala-tRNA^{Ala} (1). Enzymatic activities are reported as percentage of the wild-type activity; duplicates or triplicates were performed to determine standard errors.

Interaction analysis by EMSA: The fluorescent FAM-miHx^{Ala} was incubated with dimeric *Nbra*-CDPS^{S34A} or monomeric *Nbra*-CDPS$^{mono/S34A}$ (concentration ranging from 39 nM to 10 µM) in a buffer (20 mM sodium phosphate pH 7.5, 120 mM NaCl, 5% glycerol, 1 mM DTT). Complexes were incubated 20 min at 4°C, then 2 µL of a loading buffer containing 40% glycerol stained with Orange G dye was added to 10 µL of each mixture. Prior to the loading on a native 0.5x Tris/borate/EDTA polyacrylamide gel, a pre-migration step was achieved for 30 min at 200 V and 4°C. The migration was then performed for 60 min in the same conditions. Gels were visualized using a BioRad ChemiDoc MP Gel Imaging System. Images were processed and the fluorescence was quantified using Image Lab™ Software, version 6.1.0.07, from Bio-Rad Laboratories, Inc.

Crystallization of *Nbra*-CDPS and its variants in their apo-forms, bound to miHx^{Ala}, miHx^{Glu}, Ala-miHx^{Ala} or Ala-(amide)-miHx^{Ala}: Crystallization screenings were performed at the I2BC crystallization platform using a Mosquito-LCP robot (TTP LabTech's) and drops were visualized with a RockImager 182 or 1000 (Formulatrix). Screenings with NeXtal crystallization 96 well kits (The Classics, The Nucleix and The PEGs Suites) were performed at 4°C and 18°C. First, various concentrations of *Nbra*-CDPS, *Nbra*-CDPS^{S34A}, and untagged *Nbra*-CDPS^{S34A} were tested (from 1.5 mg/mL to 9 mg/mL) with 1.2 to 6 molar excesses of miHx^{Ala} and without miHx^{Ala}. Additional tests were carried out with complex purified by size exclusion chromatography columns (Superdex 75 10/300 column, Cytiva). Some assays were performed also by changing the ratio of the purified complex and the precipitation solution (from 1/1 to 2/1). Crystals of the *Nbra*-CDPS/miHx^{Ala} complex diffracting to 3.3 Å were obtained with the following optimized condition: 1.2 molar excess of miHx^{Ala} by monomer and vapor diffusion crystallization in tri-sodium citrate buffer (125 mM, pH 5.2), PEG3350 precipitant (from 31 to 40% w/v) and xylitol as additive (from 3 to 9%). These conditions were successfully reproduced and allowed us to crystallize the complex *Nbra*-CDPS/miHx^{Glu} in two different crystal forms that diffracted to 3.4 Å and 3.5 Å resolution. Crystals were frozen in liquid nitrogen by adding 20% glycerol and 6% xylitol for data collection at synchrotron SOLEIL, France.

Crystal structure of *Nbra*-CDPS bound to miHxs: Diffraction data were collected at 100K on PROXIMA-2A beamline. The dataset was indexed and integrated using the XDSME package (XDS Made Easier, https://github.com/legrandp/xdsme), the XDS package (2), and the CCP4 software suite (3). Crystal structure of apo *Nbra*-CDPS^{S34A} was determined by Molecular Replacement with Molrep from the CCP4 suite software (3), by using a previous crystal structure of *Nbra*-CDPS alone (PDB: 5MLQ) (4). Crystal structures of *Nbra*-CDPS/miHx^{Ala} and of *Nbra*-CDPS/miHx^{Glu} were determined by Molecular Replacement with Phaser from Phenix software package (5) using the previous apo structure.
The asymmetric unit contains seven *Nbra*-CDPS dimers: two dimers with two miHx^{Ala} bound, three dimers with one miHx^{Ala} bound and two dimers without miHx^{Ala}. For miHx^{Glu} we obtained two crystal forms. In Form I, the asymmetric unit contains a similar arrangement than the one described for *Nbra*-CDPS/miHx^{Ala} above (two dimers with two miHxs, three dimers with one miHx and two dimers with no miHx). The crystal form II contains only two *Nbra*-CDPS/miHx^{Glu} dimers with one dimer bound to two miHx^{Glu} and the other dimer without miHx (**SI Table S2**). Crystal forms I and II diffract to respectively 3.4 Å and 3.5 Å resolution

MiHx^{Ala} and miHx^{Glu} were observed in the electron density of the asymmetric unit of *Nbra*-CDPS/miHx^{Ala} and *Nbra*-CDPS/miHx^{Glu} crystals respectively. MiHxs were positioned using Coot (3) and refined with successive iterations on Refine from Phenix (5) and Buster from Global Phasing (6). We introduced in the refinement non-crystallographic symmetry (NCS). In the final models, all nucleotides from miHx^{Ala} and miHx^{Glu} were visible but residues 232 to 241 of the C-terminal extremity of *Nbra*-CDPS were not visible. The quality of the models was assessed using PDB validation service One Deep. Data collection and refinement statistics for the crystal structures are presented in **SI Table S2**.

Molecular simulation of the structure of the aminoacyl-enzyme intermediate, in which S34 is alanylated: We used the structure of the *Nbra*-CDPS in complex with miHx^{Ala} as starting point to perform MD simulations. Then the initial structure was submitted to a protocol of refinement by molecular dynamics (MD) simulation in explicit solvent. The dimeric form of the *Nbra*-CDPS was used with one miHx^{Ala} per *Nbra*-CDPS monomer. No symmetry restraints were applied during the MD simulation to double the conformational sampling during the refinement step. The initial structure was first set to the CHARMM36 topology (7) using the CHARMM software v47b1 (8). Approximate Force field parameters for the alanyl moiety were deduced from related groups parametrized in CHARMM36. Geometrical and charge parameters were checked with the Elbow module of Phenix using UHF method and AM1 level of theory (9). The initial complex was immersed in a cubic waterbox whose size was set such that the minimum distance between the solute and the edge of the box in each direction was 12 Å. This was achieved with the solvate plugin of VMD (10). The resulting hydrated complex was

neutralized with the autoionize plugin of VMD by adding sodium and chloride ions at a concentration calculated to obtain an ionic strength of 150 mM. Periodic boundary conditions were used using Particle Mesh Ewald electrostatics (grid spacing 1 Å). The cut-off for electrostatics and Lennard Jones terms was set to 12 Å and these interactions were smoothened from 10 Å to 12 Å using a switching function. The dielectric constant was set to 1.0. Langevin dynamics was used with a temperature set to 310 K. During these steps, positional restraints were applied to CA and CB atoms of *Nbra*-CDPS residue and P atoms of the $miHx^{Ala}$. The initial structure was submitted to 15000 steps minimization during which the constant on the positional restraints was decreased from 10 kcal mol^{-1} $Å^{-2}$ to 1 kcal mol^{-1} $Å^{-2}$. Then, 30106 steps MD simulation were calculated with a timestep of 2fs (60 ns) with a constant of 1 kcal mol^{-1} $Å^{-2}$ on the positional restraints. This equilibration step was followed by 250106 steps of unrestrained MD simulation (0.5 µs). MD simulations were achieved with the NAMD 2.14 CUDA software (11) on the massively parallel computer Jean-Zay HPE SGI 8600 equipped with v100 NVIDIA GPU at IDRIS (Institute for Development and Resources of Scientific computing of CNRS). Each calculation was run on 40 cores of 4 GPU using 16 Gb RAM. Trajectory analysis was achieved with ChimeraX, VMD (10), and R (R Core Team (2022), and "R: A language and environment for statistical computing. R Foundation for Statistical Computing, Vienna, Austria. URL https://www.R-project.org").

Optimization of the conditions of the stability of *Nbra*-$CDPS^{mono/S34A}$ and *Nbra*-$CDPS^{S34A}$ in solution using TSA: Thermal shift assays (TSA) were carried out on the dimeric *Nbra*-$CDPS^{S34A}$ and its monomeric variant to test and compare their respective stability and to optimize the buffer conditions. For these tests, solutions containing each protein were mixed with SYPRO Orange dye. Initial Tm measurements were performed in 115 mM Tris-HCl buffer pH 7.5, 322 mM NaCl and 5.75 % glycerol. In order to find the best conditions of stability for the two proteins, 10 pH values and 48 screening conditions were tested in duplicate, comprising a set of buffers (Tris-HCl pH 8.5, sodium potassium phosphate pH 7.5, HEPES pH 7 or MES pH 6) and adding either NaCl (from 100, 200, 300 or 500 mM), KCl (300 mM), $MgCl_2$ (10 mM) or glycerol (10%). For each condition, the mean Tm values of the duplicates were calculated. Denaturation curves were obtained after 60 min in volumes of 20 µL. We initially carried out an assay to optimize the protein concentration, which ranges typically from 2 to 10 µM. As the signal resulting from CDPS denaturation was sufficiently robust, we used a final concentration of 3.5 µM monomeric and 1.75 µM dimeric *Nbra*-$CDPS^{S34A}$ in the assays while using a 10X final concentration of SYPRO Orange dye. The screening experiments were performed using a 7900HT FAST REAL-TIME PCR and a Eppendorf epMotion 5075 pipetting robot for filling 96 wells microplates.

NMR analyses of the interaction between the monomeric variant *Nbra*-$CDPS^{mono/S34A}$ and $miHx^{Ala}$ in the presence or absence of $MgCl_2$: The interactions between *Nbra*-$CDPS^{mono/S34A}$ and the $miHx^{Ala}$ were

investigated from both the protein and RNA sides. To analyze the variations in chemical shift and intensity observed for *Nbra*-CDPS$^{mono/S34A}$ in the presence of miHx^{Ala}, we attempted to assign backbone resonances of *Nbra*-CDPS$^{mono/S34A}$ using the standard triple resonance experiments: HNCA, HN(CO)CA, HNCO, HNCACB, and HN(CO)CACB. All 3D experiments were performed as BEST (band-selective excitation short-transient) experiments (12) at proton frequencies of 800 MHz, on a ^{15}N-^{13}C-^{2}H labeled *Nbra*-CDPS$^{mono/S34A}$ sample in 100 mM phosphate sodium buffer, pH 7.4, 500 mM NaCl, 10% glycerol, 2 mM TCEP, at 303 K. These sample conditions were optimized to increase the protein lifetime at high temperature from a TSA-based buffer screening (see **SI Fig. 7**). NMR data were processed using TOPSPIN 4.1.4 software (Bruker) and analyzed using CcpNmr 2.4.2 software (http://www.ccpn.ac.uk) (13).

We also assigned the imino protons of G and U bases of miHx^{Ala} using the known assignment of a similar RNA duplex lacking the loop (14) and additional 1D ^{1}H and 2D NOESY spectra recorded with 160 ms mixing time. In the absence of $MgCl_2$, the 1D ^{1}H NMR spectrum of miHx^{Ala} showed strong and intense signals for imino protons that could be assigned to all expected base-pairs in the miHx^{Ala} helix (**SI Fig. S14b**). This demonstrates the proper folding of the helix and the subsequent protection of imino protons from solvent exchange. Three additional imino ^{1}H were also visible but broader. They were ambiguously assigned to the three bases in the loop, which suggests that these 3 bases are at least partially protected from solvent-exchange.

To investigate the binding from *Nbra*-CDPS$^{mono/S34A}$ side, we did titration by following the effect of the addition of miHx^{Ala} to a sample of 175 µM $^{15}N^{13}C$ -*Nbra*-CDPS$^{mono/S34A}$, up to a *Nbra*-CDPS$^{mono/S34A}$/miHx^{Ala} ratio of 2 molar equivalents by recording ^{1}H-^{15}N BEST-TROSY at 298K in absence of $MgCl_2$. To investigate the binding from miHx^{Ala} side, we collect ^{1}H water suppressed spectra at increasing concentration of unlabeled *Nbra*-CDPS$^{mono/S34}$ up to 2 equivalents (see **Fig. 5c**). To assess the effect of Mg^{2+} on the *Nbra*-CDPS$^{mono/S34A}$/miHx^{Ala} interaction, 16 mM $MgCl_2$ was added on the final sample of the previous titration (*ie* 2:1 ratio *Nbra*-CDPS$^{mono/S34A}$/miHx^{Ala}) and an additional ^{1}H water suppressed spectrum was collected (see **Fig. 5c**).

To assess the effect of Mg^{2+} on miHx^{Ala} structure, we titrated a 150 µM miHx^{Ala} with 0 to 8 mM of $MgCl_2$ and followed by 1D ^{1}H spectra (**SI Fig. 14b,c**). NMR spectroscopy samples were solubilized in a buffer containing 20 mM $NaHPO_4$ pH 7.3, 100 mM NaCl, 5% glycerol, 1 mM DTT, 95% H_2O / 5% D_2O. The spectra were acquired on a 950MHz AVANCE III HD Bruker spectrometer equipped with TCI cryoprobes at 283K (**SI Fig. 8**).

Cryo-EM structure of *Nbra*-CDPS^{S34A} bound to tRNA^{Ala}: *Nbra*-CDPS^{S34A} was freshly used after a two steps purification (see above). We mixed 2.7 equivalent of unacylated tRNA^{Ala} with *Nbra*-CDPS^{S34A} at 1.6 mg/mL. The *Nbra*-CDPS^{S34A}/tRNA^{Ala} was dialyzed against a phosphate buffer (15 mM sodium

phosphate, 45 mM NaCl, 5% glycerol, 1 mM DTT). The complex was deposited on a glow-discharged Quantifoil R1.2/1.3 300 mesh Copper Grid using a FEI Vitrobot at 4°C with 100% humidity and 3 s blotting time. The grids were then plunge-frozen in liquid ethane. Cryo-EM data were collected on a Glacios microscope equipped with a falcon4i camera on the Nanoimaging platform at Institut Pasteur. Details of the collection parameters can be found in **Table S3**. From the 5114 images collected, 422733 particles were picked by the Blob Picker of cryoSPARC after path motion and CTF corrections and were subjected to 2D classification, Template Picker and a second round of 2D classifications for an *ab initio* reconstruction to generate an initial 3D model (15). Several rounds of heterogeneous refinement followed by a homogeneous refinement and a final local refinement gave an improved model at 4.2 Å resolution containing 209,133 particles. Final map resolution was calculated in cryoSPARC by Fourier shell correlation at 0.143 cut-off. Cryo-EM map post-processing was performed using EMReady2 (doi: 10.1016/j.bpj.2024.11.3223), a deep-learning-based map enhancement tool. All the process is summarized schematically in **SI Fig. 13**.

SI Table 1. DNA primers used for constructing *Nbra*-CDPS variants

Variants	Mutagenic primers
Nbra-CDPS^{S34A}	see [12]
Nbra-CDPS^{mono}	F: CGTATTGATTGTCCGACCGAAGTTCTGCGTTGGGATGATGCACTGCGTCATCCGCGTTATGGTGATC<br>R: GATCACCATAACGCGGATGACGCAGTGCATCATCCCAACGCAGAACTTCGGTCGGACAATCAATACG
Nbra-CDPS$^{mono/S34A}$	F: CCGTTCTGCTGGTTGCCGTTGGTGCAGATTATC<br>R: GATAATCTGCACCAACGGCAACCAGCAGAACGG
Nbra-CDPS without His-tag	F: CGTGAAGAACAGAGATCTTAACACCATCACCATCACTAAGC<br>R: GCTTAGTGATGGTGATGGTGTTAAGATCTCTGTTCTTCACG
Nbra-CDPS^{L32G}	F: GGTCGTAGCACCGTTCTGGGTGTTAGCGTTGGTGCAG<br>R: CTGCACCAACGCTAACACCCAGAACGGTGCTACGACC
Nbra-CDPS^{L32A}	F: GGTCGTAGCACCGTTCTGGCGGTTAGCGTTGGTGCAGATTATC<br>R: GATAATCTGCACCAACGCTAACCGCCAGAACGGTGCTACGACC
Nbra-CDPS^{S199L}	F: GATTTTGTTATTTATCCGCAGCGTATTCTCGCAGCAATGGGTCGTACC<br>R: GGTACGACCCATTGCTGCGAGAATACGCTGCGGATAAATAACAAAATC

SI Table 2. Data collection and refinement statistics

(Pdb code)	***Nbra*-CDPS^{S34A}**<br>(9I5M)	***Nbra*-CDPS/miHx^{Ala}**<br>(9IAJ)	***Nbra*-CDPS /miHx^{Glu}**<br>form II<br>(9IAL)	***Nbra*-CDPS /miHx^{Glu}**<br>form I<br>(9IAK)	***Nbra*-CDPS^{S34A}/**<br>**Ala-(amide)-miHx^{Ala}**<br>(9IAM)
Data collection<br>Space group	$P\ 2_1\ 2_1\ 2$	$P\ 2_1\ 2_1\ 2_1$	$P\ 2_1\ 2_1\ 2_1$	$P\ 2_1\ 2_1\ 2_1$	$P\ 2_1\ 2_1\ 2_1$
Cell dimensions<br>a, b, c (Å)<br>α, β, γ (°)	85.54, 87.80, 67.72<br>90, 90, 90	105.03, 170.32, 321.12<br>90, 90, 90	96.48, 111.09, 163.95<br>90, 90, 90	106.37, 170.50 323.93<br>90, 90, 90	104.97, 170.03, 319.39<br>90, 90, 90
Resolution (Å)<br>Total number of observations<br>Total number unique	45.38-1.73<br>(1.84- 1.73)[a]<br>568,188<br>(29,346)<br>43588 (2222)	49.35- 3.29<br>(3.57- 3.29)[a]<br>902,920 (45,167)<br>65,928 (3,297)	49.04-3.40<br>(3.70- 3.40)[a]<br>275,306 (13,369)<br>18,334 (917)	48.45 - 3.51<br>(3.56 - 3.52) [a]<br>759,526 (39,815)<br>55,511 (2,923)	49.3-3.61<br>(4.06- 3.61)[a]<br>460,642 (32,349)<br>45,502 (2,528)
I / σ(I)	14.3 (1.3)	7.3 (1.5)	7.7 (1.5)	7.4 (1.9)	5.6 (1.6)
Completeness ellipsoidal (%)<br>Completeness spherical (%)<br>$CC_{1/2}$	89.3 (55.5)<br>81.6 (26.5)<br>0.997 (0.375)	95.1 (75.2)<br>74.6 (17.6)<br>0.993 (0.412)	94.4 (66.6)<br>73.7 (16.6)<br>0.997 (0.699)	94.7 (68.8)<br>75.3 (16.8)<br>0.993 (0.591)	92.8 (63.6)<br>68.5 (13.0)<br>0.993 (0.573)
R_{work}/R_{free}	0.211/0.234	0.207/0.243	0.204/0.247	0.218/0.246	0.224/0.243
No. atoms<br>Protein<br>Nucleic acid<br>Ligand/ion<br>Water	4,111<br>3,662<br>0<br>0<br>449	28,881<br>25,499<br>3,262<br>120<br>0	8,253<br>7,284<br>934<br>35<br>0	28,371<br>25,494<br>2,802<br>75<br>0	28,414<br>25,618<br>2,796<br>0<br>0
Wilson B-factor ($Å^2$)	21.6	112.8	79.6	93.4	89.3
Average B, all atoms ($Å^2$)	27	126	98	111.0	101.0
R.m.s. deviations<br>Bond lengths (Å)<br>Bond angles (°)	0.008<br>1.138	0.003<br>0.751	0.007<br>1.28	0.004<br>0.84	0.004<br>0.692

The data were anisotropic and were truncated using STARANISO https://staraniso.globalphasing.org/cgi-bin/staraniso.cgi

[a] Values in parentheses are for highest-resolution shell.

SI Table 3. Cryo-EM data collection and refinement statistics

**Data collection and processing**	*Nbra*-CDPS-tRNA^{Ala}
Detector	
Magnification	190k
Voltage (kV)	200
Flux on detector (e/pix/sec)	7.47
Electron exposure on sample (e–/Å^2)	50
Target defocus range (µm)	(-2.75) – (-0.7)
Calibrated pixel size (Å)	0.76
Symmetry imposed	C1
Extraction box size (pixels)	160
Initial particle images (no.)	159,6771
Final particle images (no.)	209,133
**Refinement**	
Map resolution at FSC=0.143 (Å)*	4.22
Model composition	
Non-hydrogen atoms	5271
Protein residues	452
Nucleotide	76
B factor (Å^2)	
Protein	102.42
RNA	196.82
R.m.s deviations	
Bond lengths (Å)	0.005
Bond angles (°)	1.494
Validation	
Molprobity score	2.90
Clashscore	31.68
Poor rotamers (%)	0
Ramachandran plot	
Favored (%)	60.71
Allowed (%)	31.25
Outliers (%)	8.4

SI Table 4. Interactions between *Nbra*-CDPS and miHx^{Ala}

Arm region

miHx		atom	*Nbra*-CDPS	atom	interaction
A73			no contact		
C74	base	O2	Y39	OH	hyd bond
C74	base	N3	Y39	OH	hyd bond
C75	phosphate	PO4	R156	ammonium	salt bridge
C75	base		R152	ammonium	PiCation
C75	ribose	O2'	Q196	OE1	hyd bond
C75	base	N3	Q196	NE2	hyd bond
A76	phosphate	PO4	R152	ammonium	salt bridge
A76	phosphate	PO4	R156	ammonium	salt bridge
A76	ribose	O4'	R152	ammonium	hyd bond
A76	base	N6	N72	OD1	hyd bond
A76	base	N7	N72	ND2	hyd bond
A76	ribose	O3'	Y194	OH	hyd bond
A76	ribose	O2'	Y172	OH	hyd bond
A76	base		Y172	ring	stacking
A76	base		F149	ring	stacking

Helix region

miHx		atom	*Nbra*-CDPS	atom	interaction
G1	phosphate	PO4	K227	ammonium	salt bridge
G1	phosphate	PO4	K44	ammonium	salt bridge
G1	phosphate	PO4	H11	NE2	salt bridge
G2	phosphate	PO4	K227	ammonium	salt bridge
G3	base	O6	K226	ammonium	hyd bond
G4	base	O6	K226	ammonium	hyd bond
A67	phosphate	PO4	R228	ammonium	salt bridge
A67	phosphate	PO4	K12	ammonium	salt bridge

G68	phosphate	PO4	K12	ammonium	salt bridge
C69	phosphate	PO4	S19	NH	hyd bond
C69	phosphate	PO4	R18	ammonium	salt bridge
C69	phosphate	PO4	R224	ammonium	salt bridge
U70	phosphate	PO4	R224	ammonium	salt bridge

SI Table 5. Interactions between *Nbra*-CDPS and miHx^{Glu} (Forme I)

Arm region

miHx		atom	*Nbra*-CDPS	atom	interaction
A73			no contact		
C74	base	O2	Y39	OH	hyd bond
C74	base	N3	Y39	OH	hyd bond
C75	phosphate	PO4	R156	ammonium	salt bridge
C75	phosphate	PO4	R152	ammonium	salt bridge
C75	base	O2	Y39	OH	hyd bond
A76	Base	N6	C169	SG	hyd bond
A76	phosphate	PO4	R156	ammonium	salt bridge
A76	ribose	O3’	Y194	OH	hyd bond
A76	ribose	O2’	Y194	OH	hyd bond
A76	base	N6	N72	OD1	hyd bond
A76	base		Y172	ring	stacking
A76	base		F149	ring	stacking

Helix region

miHx		atom	*Nbra*-CDPS	atom	interaction
G1	phosphate	PO4	H11	NE2	salt bridge
G1	phosphate	PO4	K44	ammonium	salt bridge
G1	phosphate	PO4	K227	ammonium	salt bridge
U2	phosphate	PO4	K227	ammonium	salt bridge
G68	base	O6	K226	ammonium	hyd bond
G69	base	O6	K226	ammonium	hyd bond
G67	base	O6	K229	ammonium	hyd bond
G67	phosphate	PO4	K12	ammonium	salt bridge
G67	phosphate	PO4	R228	ammonium	salt bridge
G69	phosphate	PO4	S19	OH	hyd bond
G69	phosphate	PO4	R224	ammonium	salt bridge
G70	phosphate	PO4	R224	ammonium	salt bridge

SI Table 6. Interactions between *Nbra*-CDPS and Ala-(amide)-miHx^{Ala}

Arm region

miHx		atom	*Nbra*-CDPS	atom	interaction
A73			no contact		
C74	base	O2	Y39	OH	hyd bond
C74	base	N3	Y39	OH	hyd bond
C74	base	N4	Q196	OE1	hyd bond
C75	ribose	O4’	Y39	OH	hyd bond
A76	phosphate	PO4	R152	ammonium	salt bridge
A76	phosphate	PO4	R156	ammonium	salt bridge
A76	ribose	O2'	Y194	OH	hyd bond
A76	ribose	O2'	Y172	OH	hyd bond
A76	base	N6	C169	SG	hyd bond
A76	base	N6	C169	O	hyd bond
A76	base		F149	ring	stacking
A76	base		Y172	ring	stacking
Alanyl	carbonyl	O	Q69	OE1	hyd bond
Alanyl	carbonyl	O	N72	ND2	hyd bond

References

1. Canu, N., Tellier, C., Babin, M., Thai, R., Ajel, I., Seguin, J., Cinquin, O., Vinck, R., Moutiez, M., Belin, P. *et al.* (2020) Flexizyme-aminoacylated shortened tRNAs demonstrate that only the aminoacylated acceptor arms of the two tRNA substrates are required for cyclodipeptide synthase activity. *Nucleic Acids Res*, **48**, 11615-11625.
2. Kabsch, W. (2010) Xds. *Acta Crystallogr D Biol Crystallogr*, **66**, 125-132.
3. Winn, M.D., Ballard, C.C., Cowtan, K.D., Dodson, E.J., Emsley, P., Evans, P.R., Keegan, R.M., Krissinel, E.B., Leslie, A.G., McCoy, A. *et al.* (2011) Overview of the CCP4 suite and current developments. *Acta Crystallogr D Biol Crystallogr*, **67**, 235-242.
4. Bourgeois, G., Seguin, J., Babin, M., Belin, P., Moutiez, M., Mechulam, Y., Gondry, M. and Schmitt, E. (2018) Structural basis for partition of the cyclodipeptide synthases into two subfamilies. *J Struct Biol*, **203**, 17-26.
5. Afonine, P.V., Grosse-Kunstleve, R.W., Echols, N., Headd, J.J., Moriarty, N.W., Mustyakimov, M., Terwilliger, T.C., Urzhumtsev, A., Zwart, P.H. and Adams, P.D. (2012) Towards automated crystallographic structure refinement with phenix.refine. *Acta Crystallogr D Biol Crystallogr*, **68**, 352-367.
6. Smart, O.S., Womack, T.O., Flensburg, C., Keller, P., Paciorek, W., Sharff, A., Vonrhein, C. and Bricogne, G. (2012) Exploiting structure similarity in refinement: automated NCS and target-structure restraints in BUSTER. *Acta Crystallographica Section D*, **68**, 368-380.
7. Huang, J. and MacKerell, A.D., Jr. (2013) CHARMM36 all-atom additive protein force field: validation based on comparison to NMR data. *J Comput Chem*, **34**, 2135-2145.
8. Brooks, B.R., Brooks, C.L., 3rd, Mackerell, A.D., Jr., Nilsson, L., Petrella, R.J., Roux, B., Won, Y., Archontis, G., Bartels, C., Boresch, S. *et al.* (2009) CHARMM: the biomolecular simulation program. *J Comput Chem*, **30**, 1545-1614.
9. Moriarty, N.W., Grosse-Kunstleve, R.W. and Adams, P.D. (2009) electronic Ligand Builder and Optimization Workbench (eLBOW): a tool for ligand coordinate and restraint generation. *Acta Crystallogr D Biol Crystallogr*, **65**, 1074-1080.
10. Humphrey, W., Dalke, A. and Schulten, K. (1996) VMD: visual molecular dynamics. *J Mol Graph*, **14**, 33-38, 27-38.
11. Phillips, J.C., Hardy, D.J., Maia, J.D.C., Stone, J.E., Ribeiro, J.V., Bernardi, R.C., Buch, R., Fiorin, G., Hénin, J., Jiang, W. *et al.* (2020) Scalable molecular dynamics on CPU and GPU architectures with NAMD. *J Chem Phys*, **153**, 044130.
12. Lescop, E., Schanda, P. and Brutscher, B. (2007) A set of BEST triple-resonance experiments for time-optimized protein resonance assignment. *J Magn Reson*, **187**, 163-169.
13. Vranken, W.F., Boucher, W., Stevens, T.J., Fogh, R.H., Pajon, A., Llinas, M., Ulrich, E.L., Markley, J.L., Ionides, J. and Laue, E.D. (2005) The CCPN data model for NMR spectroscopy: development of a software pipeline. *Proteins*, **59**, 687-696.
14. Limmer, S., Hofmann, H.P., Ott, G. and Sprinzl, M. (1993) The 3'-terminal end (NCCA) of tRNA determines the structure and stability of the aminoacyl acceptor stem. *Proc Natl Acad Sci U S A*, **90**, 6199-6202.
15. Punjani, A., Rubinstein, J.L., Fleet, D.J. and Brubaker, M.A. (2017) cryoSPARC: algorithms for rapid unsupervised cryo-EM structure determination. *Nat Methods*, **14**, 290-296.
