# Supplementary material for: The tRNA moieties of both aminoacyl-tRNA substrates of a cyclodipeptide synthase share a common binding site, as revealed by RNA microhelices mimicking tRNA acceptor arms
Source: Nucleic Acids Res. 2026 Apr 7;54(6):gkag307. doi: 10.1093/nar/gkag307 (PMC13062777; doi:10.1093/nar/gkag307)
Supplement: gkag307_Supplemental_Files [file gkag307_supplemental_files.zip › Supplementary Figures.pdf]

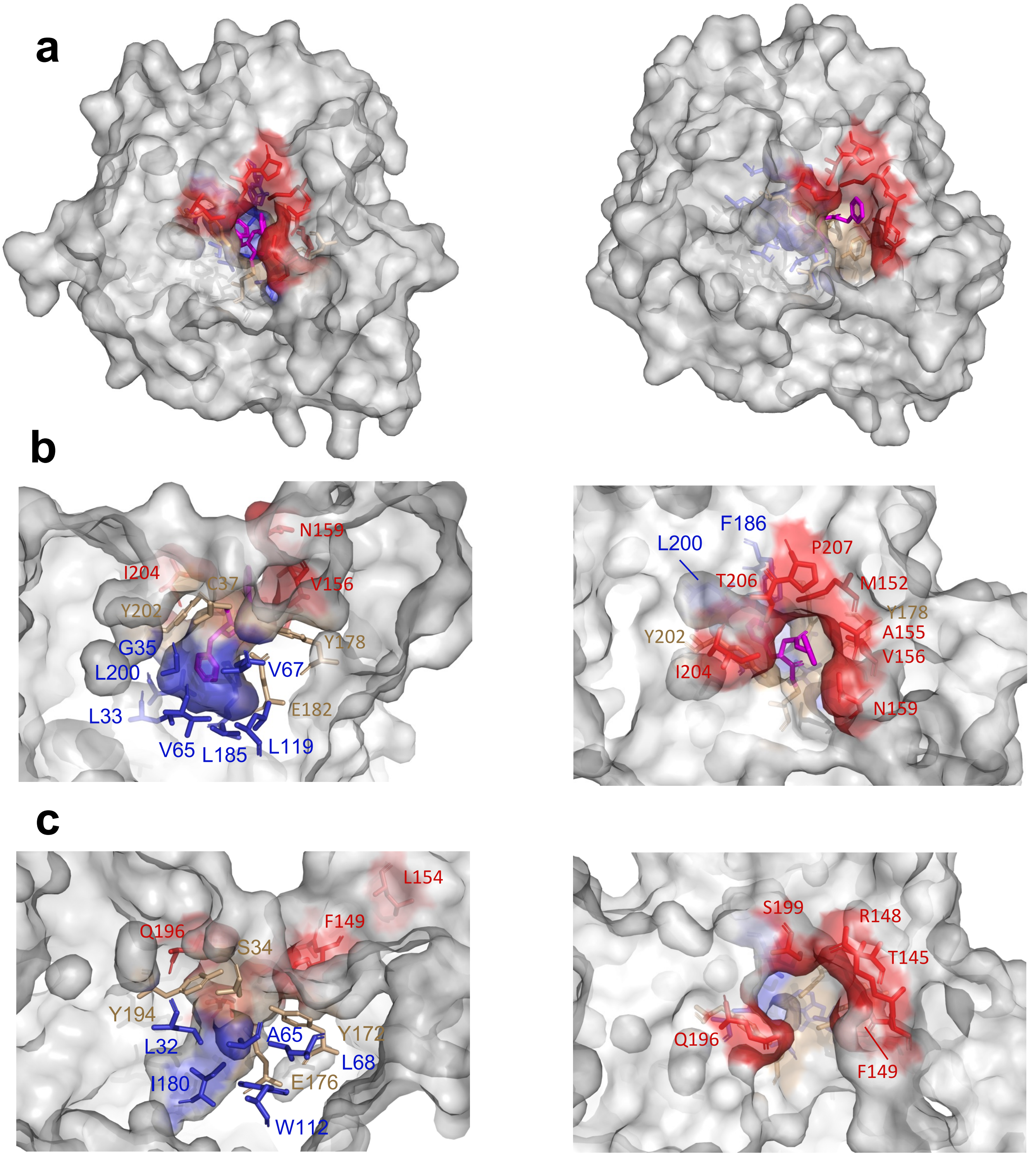

Supplementary Fig. 1: Regions of CDPSs that accommodate the aminoacyl moieties of the first substrate (P1 pocket, in blue) and the second substrate (C2 cleft, in red); the four conserved catalytic residues are in wheat. The left and right views are similar but slightly reoriented to highlight the C2 regions and the ligand. a) Overall view of *Snou*-CDPS (in grey) bound to a dipeptide analogue (in magenta) (structure of *Snou*-CDPS S37C bound to ZPK (N-carbobenzyloxy-L-Phe-methyl ketone) (PDB: 4Q24) (Moutiez et al, 2014, Nat Comm, doi: 10.1038/ncomms6141). b) Close-up view of the P1 pocket composed of 8 residues (left) and C2 cleft composed of 7 residues (right) of *Snou*-CDPS. c) Close-up view of the P1 pocket composed of 5 residues (left) and C2 composed of 5 residues (right) of *Nbra*-CDPS, obtained by superimposing the structures of *Snou*-CDPS (4Q24) and *Nbra*-CDPS (5MLQ).

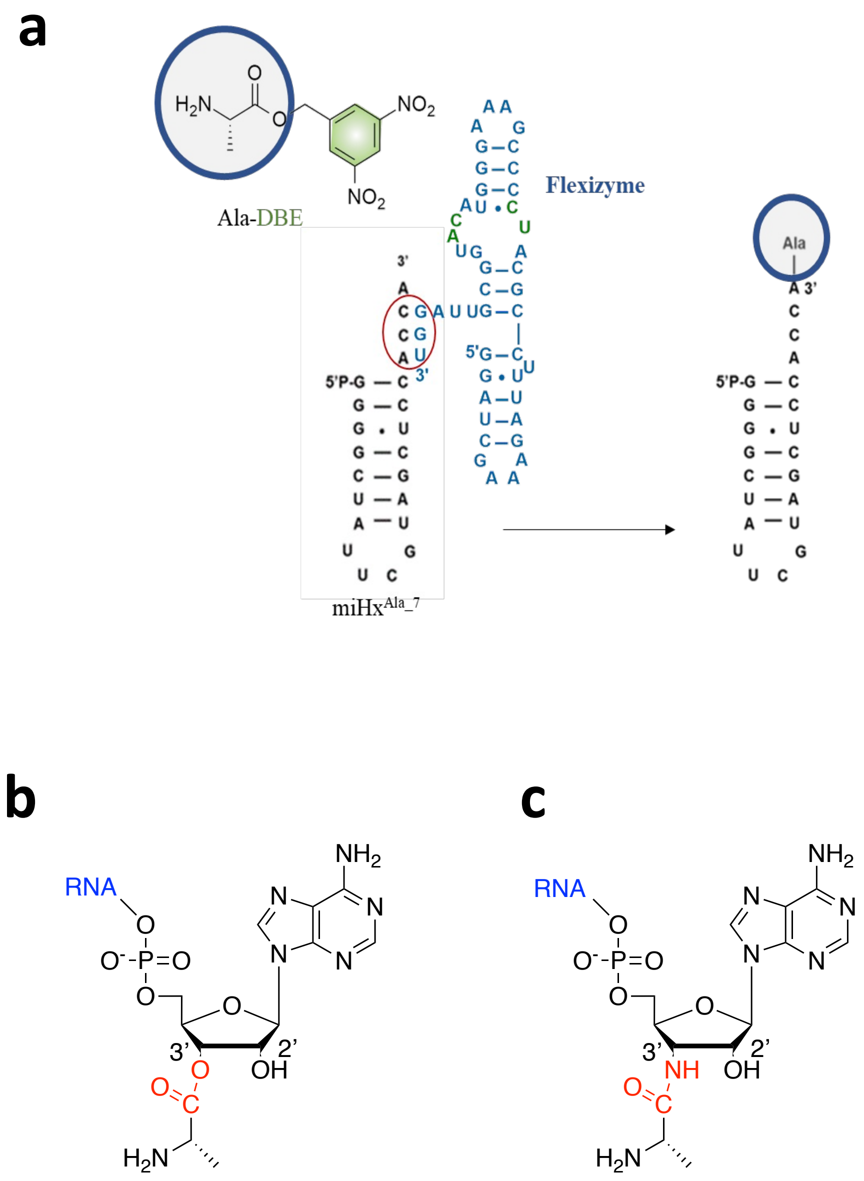

Supplementary Fig. 2: Alanylation of miHx^{Ala} catalyzed by Fxs. (a) The dFx variant, shown in light blue, uses a miHx^{Ala} and an activated amino acid, Ala-DBE, as substrates The 3′-terminal end of miHx and dFx interact, after which the alanyl moiety is transferred onto the terminal adenosine of miHx. Attachment of the alanyl moiety produces (b) Ala-miHx^{Ala}, where Ala forms an ester bond with the 3′-hydroxyl of A^{76}, and (c) Ala-(amide)-miHx^{Ala}, where Ala forms an amide bond with the 3′-amino group of A^{76}.

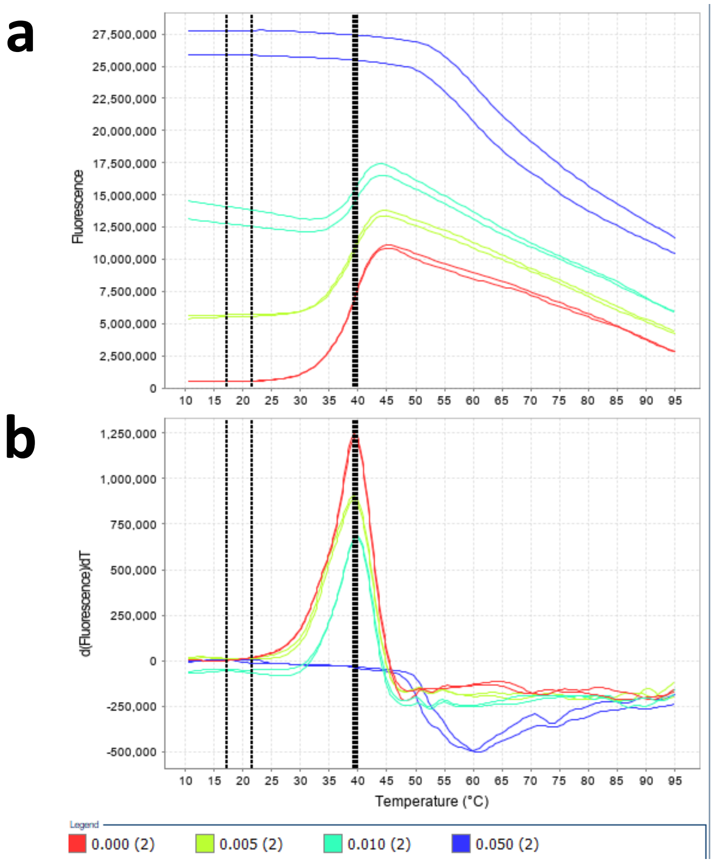

Supplementary Fig. 3: Influence of Tween 20 on *Nbra*-CDPS stability and activity. (a-b) Protein stability was assessed using thermal shift assay (TSA) in 20 mM sodium phosphate pH 7.3, 100 mM NaCl. Direct TSA measurements at Tween 20 concentration of 0.05% were not possible due to excessive signal noise. To evaluate the effect of detergent, *Nbra*-CDPS was incubated in 0.05% Tween 20 during 30 min, then diluted prior to analysis. No difference in TSA-derived melting temperature were observed between 0, 0.005% and 0.01 % Tween 20-indicating that Tween 20 does not affect *Nbra*-CDPS stability under these conditions. (a) Normalized TSA melting curves (b) derivative melting curves. The half-denaturation temperatures (Tm) of *Nbra*-CDPS$^{mono/S34A}$ is 39 °C (vertical black dashed line).

Regarding the influence of Tween 20 on enzymatic activity, it could not be evaluated. Indeed, activity measurements rely on LC–MS analysis, and Tween 20 was deliberately excluded from reaction mixtures, as its presence would cause persistent contamination of the LC column and MS source, thereby compromising both the sensitivity and the reproducibility of the analyses.

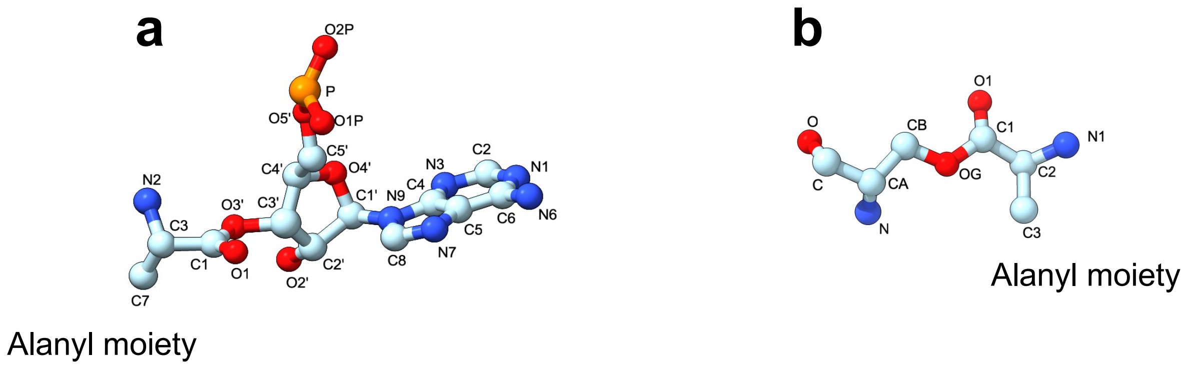

Supplementary Fig. 4: Structure of alanylated A^{76} and S34 shown in ball-and-stick representation, coloured by atom type in light blue. Atom names are indicated. (a) The alanyl moiety is linked to the O3′ atom of the ribose of A^{76} (AO3′-miHx^{Ala}). (b) The alanyl moiety is linked to S34, forming the alanyl-enzyme intermediate (SEA).

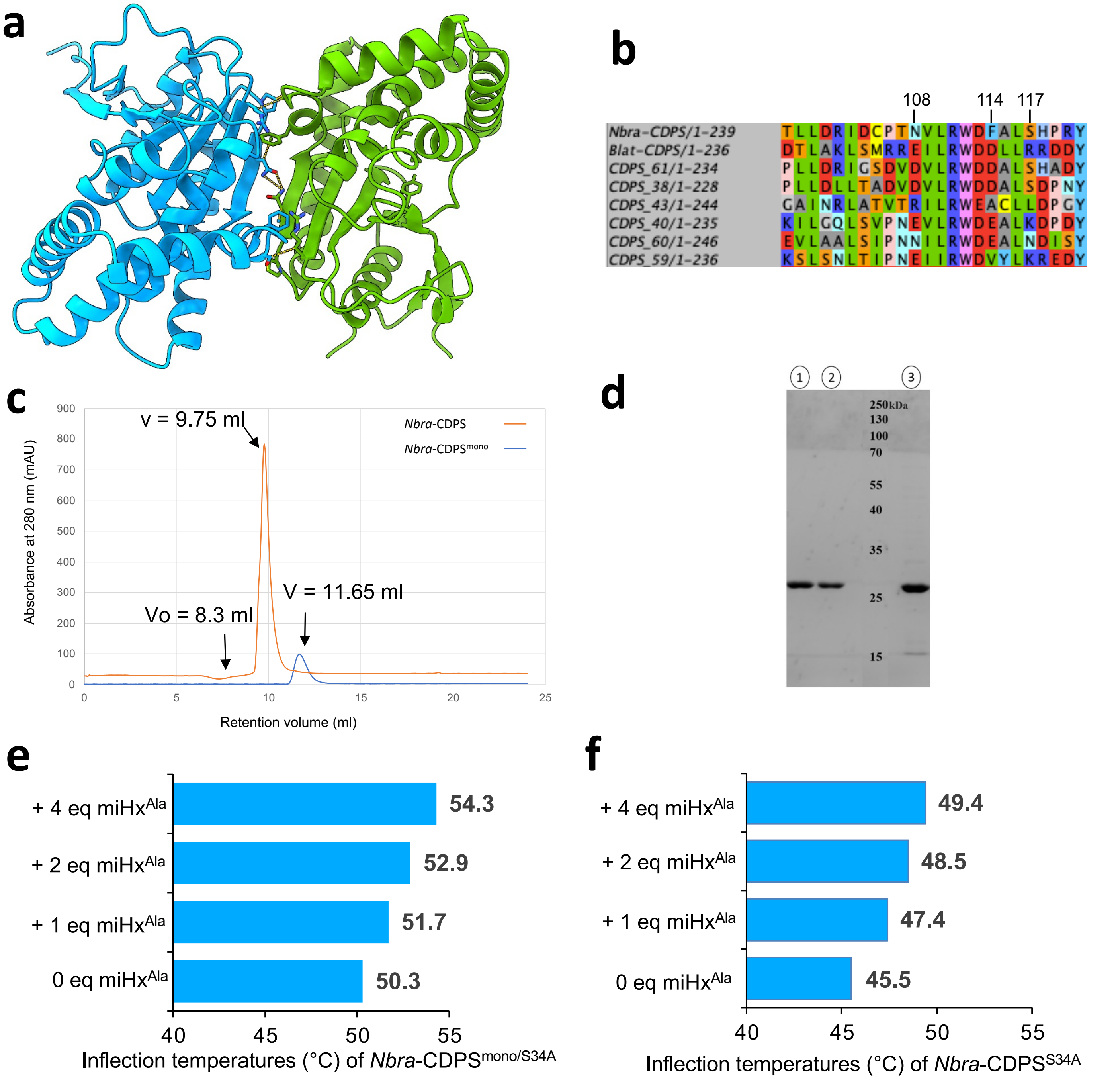

Supplementary Fig. 5: Characterization of *Nbra*-CDPS and its variants. (a) Structure of *Nbra*-CDPS (PDB: 5MLQ). (b) Multiple sequence for the eight CDPSs shown to synthesize cAE (Jacques et al 2015, Nat Chem Biol, doi: 10.1038/nchembio.1868). The N108, F114 and S117 residues in the *Nbra*-CDPS sequence correspond respectively to E, D and R in the monomeric *Blat*-CDPS (previously named as CDPS 37). (c) Size exclusion chromatography of *Nbra*-CDPS and *Nbra*-CDPS^{mono}. UV traces at 280 nm are shown in orange and blue for the wild-type enzyme and its monomeric variant, respectively. The elution volumes correspond to apparent molecular masses of 38 kDa and 19 kDa compared with expected masses of 57 kDa and 28 kDa for the dimeric and monomeric forms. The analysis was performed on a Superdex 75 10/300 column equilibrated in phosphate buffer pH 7.5, at a flow rate of 0.4 mL/min. (d) SDS-PAGE analysis of purified *Nbra*-CDPS and its variants: lane 1, *Nbra*-CDPS$^{mono/S34A}$; lane 2, *Nbra*-CDPS^{S34A}; lane 3, non-tagged *Nbra*-CDPS^{S34A}. (e-f) Thermal stability of (e) *Nbra*-CDPS$^{mono/S34A}$ and (f) *Nbra*-CDPS^{S34A} measured by nanoDSF (Ti, Inflection temperature). Line 1: no ligand; Lines 2 to 4, Ti in the presence of miHx^{Ala} at 1, 2 and 4 equivalent of *Nbra*-CDPS$^{mono/S34A}$ or *Nbra*-CDPS^{S34A} concentration.

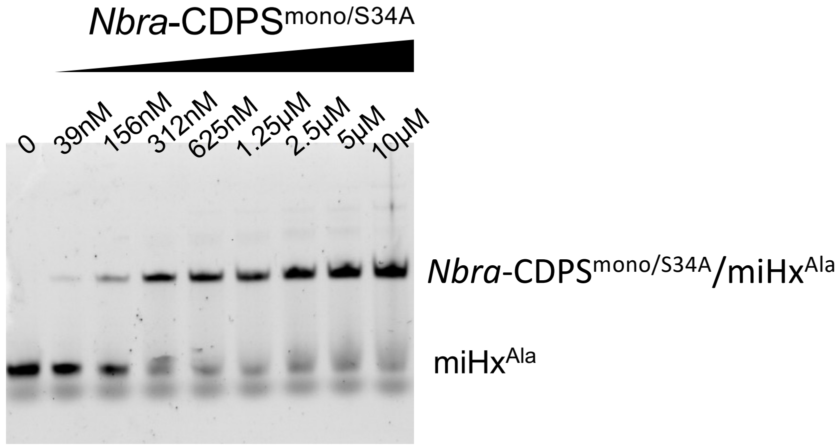

Supplementary Fig. 6: Gel shift assay of the *Nbra*-CDPS$^{mono/S34A}$ interaction with a fluorescently labelled FAM-miHx^{Ala}. The EMSA shows interactions starting about 40nM.

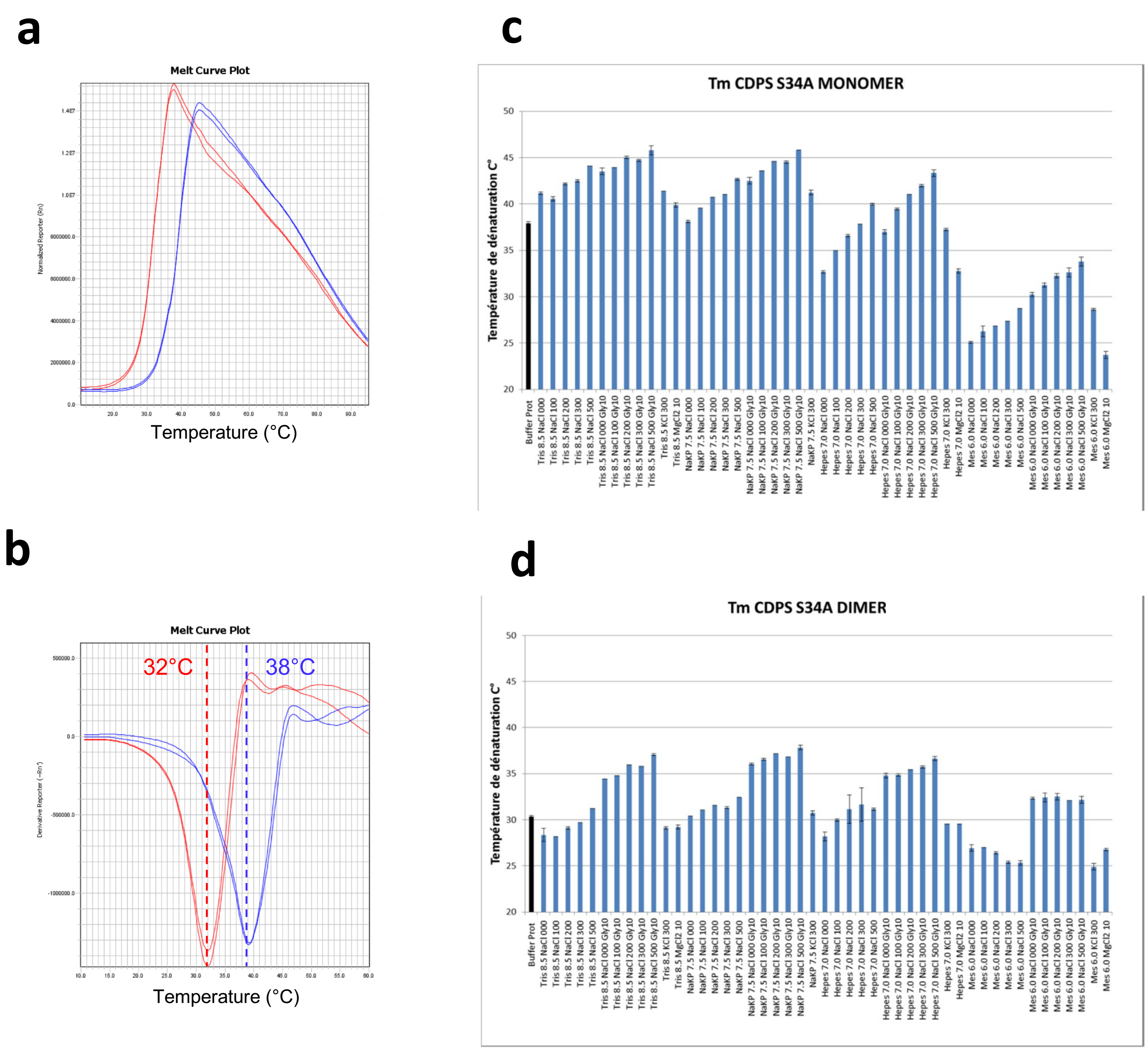

Supplementary Fig. 7: Optimization of the conditions of the stability of *Nbra*-CDPS$^{mono/S34A}$ and *Nbra*-CDPS^{S34A} in solution using TSA. Comparison of monomer *Nbra*-CDPS$^{mono/S34A}$ (blue) and dimer *Nbra*-CDPS^{S34A} (red) stability using TSA. (a) TSA normalized melting curves and (b) derivative melting curves comparing the stabilities under the same conditions: 115 mM Tris-HCl buffer pH7.5; 322 mM NaCl and 5.75% glycerol. The half-denaturation temperatures (Tm) were found to be 38°C for *Nbra*-CDPS$^{mono/S34A}$ (vertical blue dotted line), and 32°C for *Nbra*-CDPS^{S34A} (vertical red dotted line). TSA-derived denaturation temperature (Tm) of monomeric (c) and dimeric *Nbra*-CDPS^{S34A} (d) for a range of buffers. The highest Tm is recorded for *Nbra*-CDPS$^{mono/S34A}$ at 46°C with 500 mM NaCl and 10% glycerol added to either Tris-HCl buffer pH 8.5 or sodium potassium phosphate buffer pH 7.5. Similarly to the results mentioned above for the monomer, the addition of 500 mM NaCl and 10% glycerol to sodium potassium phosphate buffer pH 7.5 increases the stability of the dimer, with a Tm of 37°C, confirming the observation done on the monomeric form.

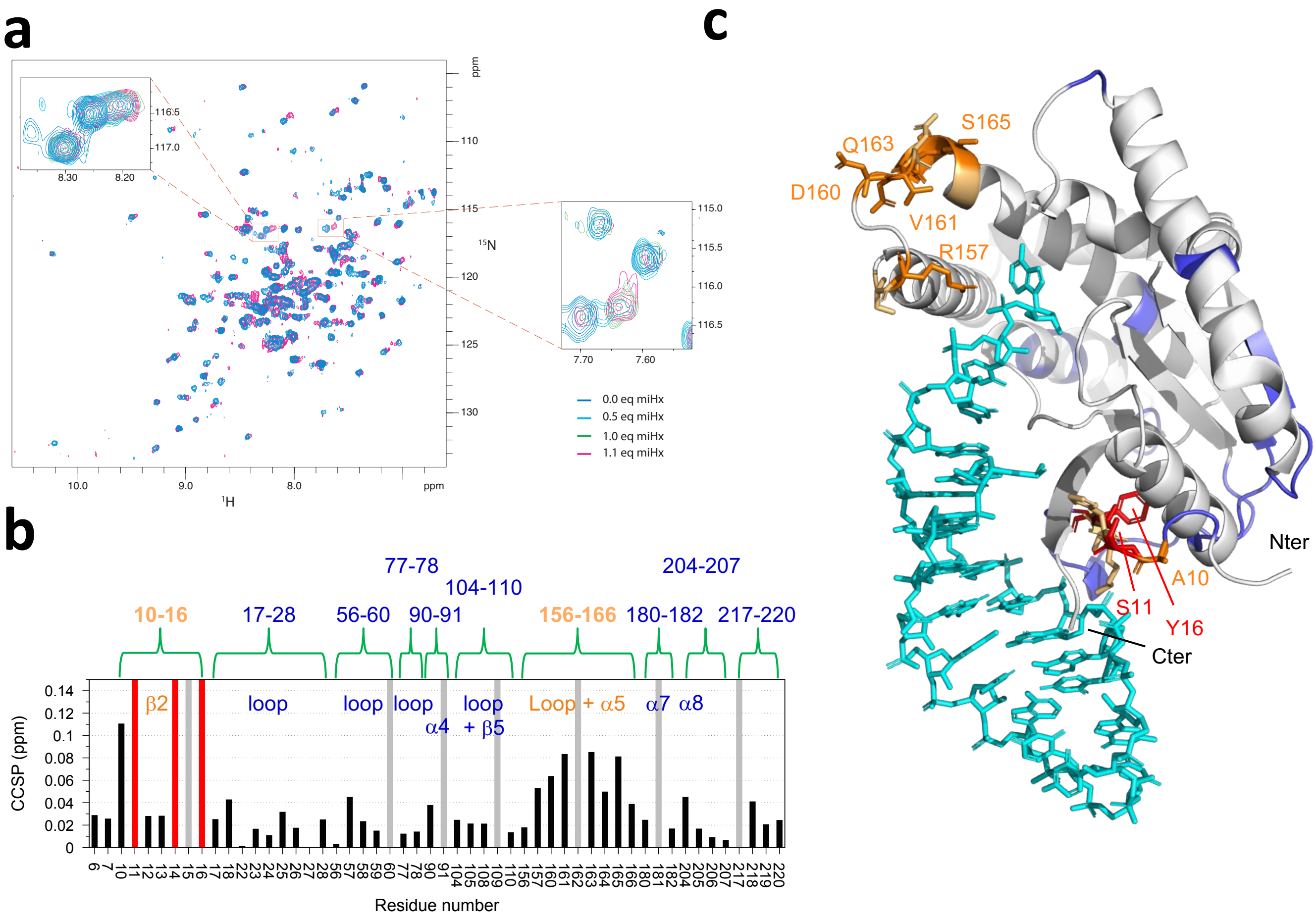

Supplementary Fig. 8: Characterization of the interaction between *Nbra*-CDPS$^{mono/S34A}$ and miHx^{Ala} using NMR spectroscopy. (a) To investigate the binding of *Nbra*-CDPS$^{mono /S34A}$ and miHx^{Ala}, we did titration by following the effect of the addition of miHx^{Ala} to a sample of 175 µM ^{15}N^{13}C -*Nbra*-CDPS$^{mono/S34A}$, up to a miHx^{Ala}/*Nbra*-CDPS$^{mono/S34A}$ ratio of 1.1, by recording ^{1}H-^{15}N BEST-TROSY at 298K on a Bruker Avance III 800 MHz spectrometer equipped with a ^{1}H-^{13}C-^{15}N triple resonance TCI cryoprobe with a Z gradient. The figure shows the superimposition of the BEST-TROSY ^{1}H-^{15}N spectra recorded in the absence of miHx^{Ala} (dark blue), and in the presence of 0.5, 1.0 and 1.1 molar equivalent of miHx^{Ala} (in light blue, green and red, respectively). (b) Analysis of the ^{1}H-^{15}N Combined Chemical Shift Perturbations (CCSP = [(ΔδH)^2 + (ΔδN)^2*0.14)]^1/2) calculated from the ^{1}H-^{15}N BEST-TROSY spectra of *Nbra*-CDPS$^{mono/S34A}$ recorded at 1.1 and 0 molar concentrations of miHx^{Ala}. The analysis could be done for the assigned cross-peaks of the CDPS, that mostly correspond to flexible loops. The chemical shift changes are quite significant (CCSP > 0.05 ppm) for several residues, including A10 and 157-165. Several other residues disappear after the addition of miHxAla due to intermediate or slow exchange, and these are represented by vertical red lines (S11, A14, Y16). It was not possible to determine the chemical shift variations for some residues because their cross-peaks were superimposed on those of others after the addition of miHxAla (vertical grey lines). On the x-axis, only the numbers of the residues assigned are shown. (c) Residues for which significant chemical shift perturbations (> 0.05 ppm) have been observed are highlighted on the *Nbra*-CDPS$^{mono/S34A}$/miHx^{Ala} crystal structure as dark orange sticks, and those that have disappeared are shown in red. The other residues of *Nbra*-CDPS$^{mono/S34A}$ that have been attributed, but which do not show variations in chemical shifts in the presence of miHx^{Ala}, are shown in blue.

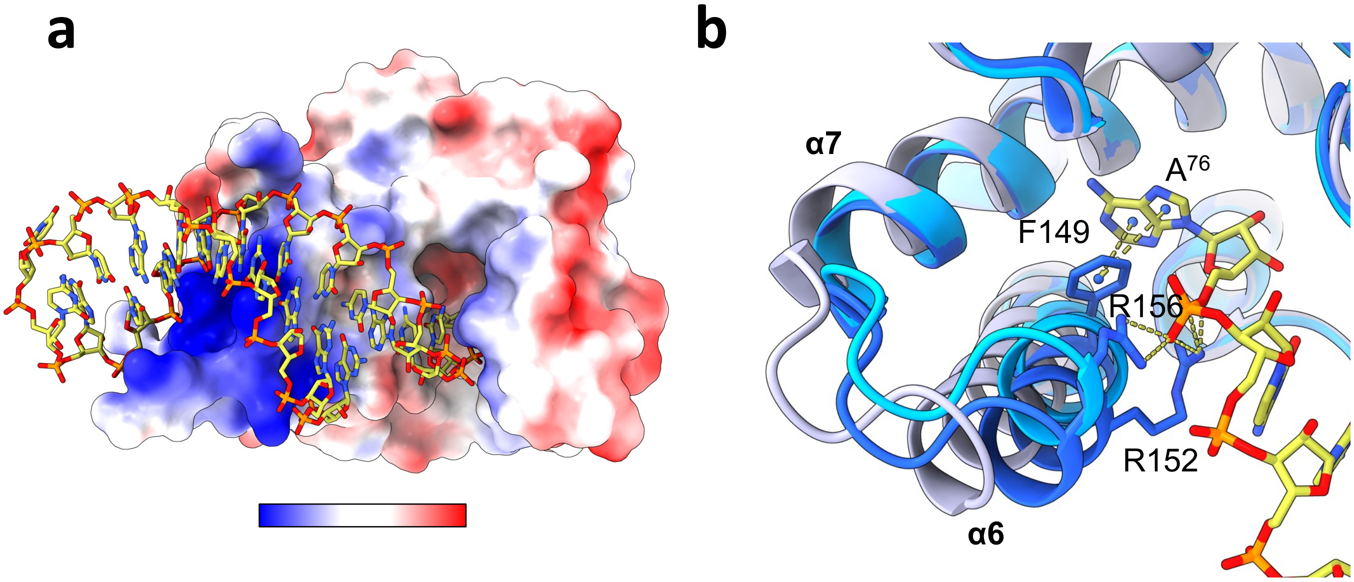

Supplementary Fig. 9: Characterization of *Nbra*-CDPS bound to a miHx^{Ala}. (a) Electrostatic surface potential view of *Nbra*-CDPS colored by electrostatic potentials (blue surfaces represent positively charged residues, red for negatively charged residues and white for neutral residues). (b) Superposition of *Nbra*-CDPS/miHx^{Ala} (PDB: 9IAM, 3.6 Å in blue) on apo-*Nbra*-CDPS^{S34A} (PDB: 9I5M, 1.7 Å in cyan) and the previously reported apo structure (PDB: 5MLQ, 3.2 Å in lavender) . Zoom that shows the rearrangement of the helix α6, specifically residues F149, R152 and R156, stabilizing the complex through hydrogen bonds and π-stacking.

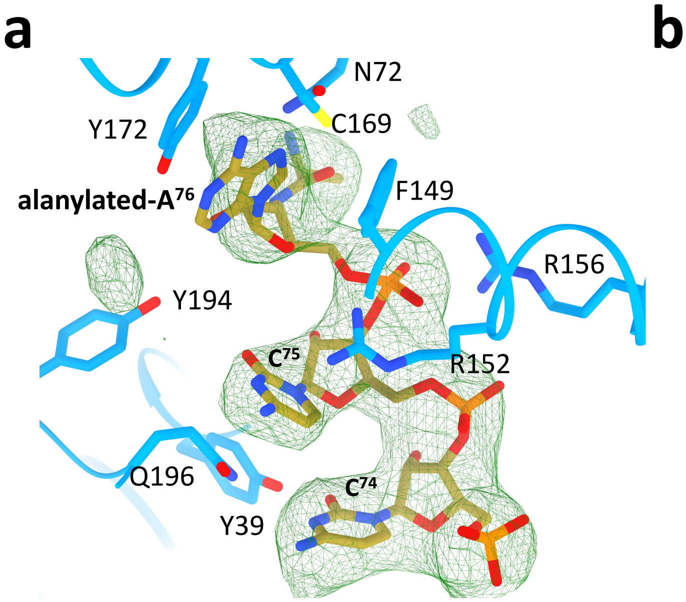

Supplementary Fig. 10: The OMIT maps of C^{74}, C^{75} and the alanylated A^{76} in the asymmetric unit. The maps are shown as green mesh contoured at 3.0 σ. Interacting protein residues are represented as described in **Fig. 4**. The OMIT maps were calculated using phenix.polder (Liebschner et al, 2017, Acta Crystallogr D Struct Biol, doi: 10.1107/S2059798316018210) for the corresponding $C^{74}C^{75}A^{76}$–Ala in each of the five miHxs present in the asymmetric unit. (a) mFo–DFc OMITmap for the $C^{74}C^{75}A^{76}$– Ala. (b) Close-up view of the alanylated A^{76}.

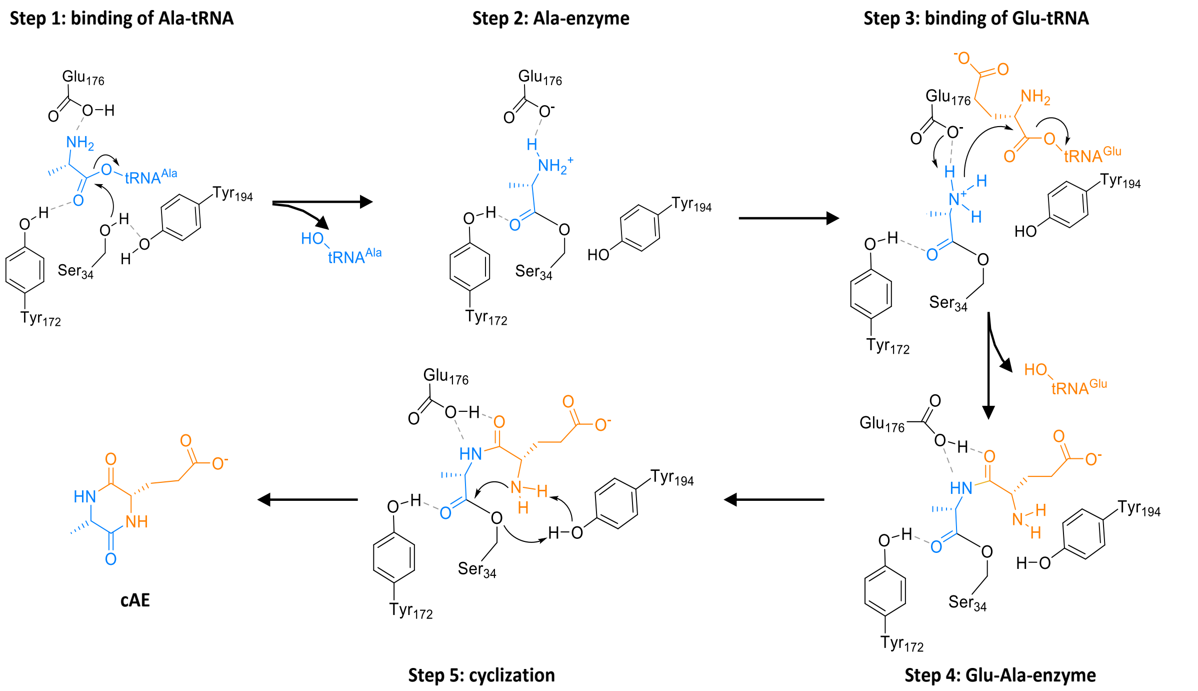

Supplementary Fig. 11: Mechanism proposed for cAE synthesis by *Nbra*-CDPS. The first and second substrates are highlighted in blue and orange, respectively. Step 2 corresponds to the formation of the alanylated enzyme intermediate, which is modeled in the present study. Possible interactions are indicated by dashed lines.

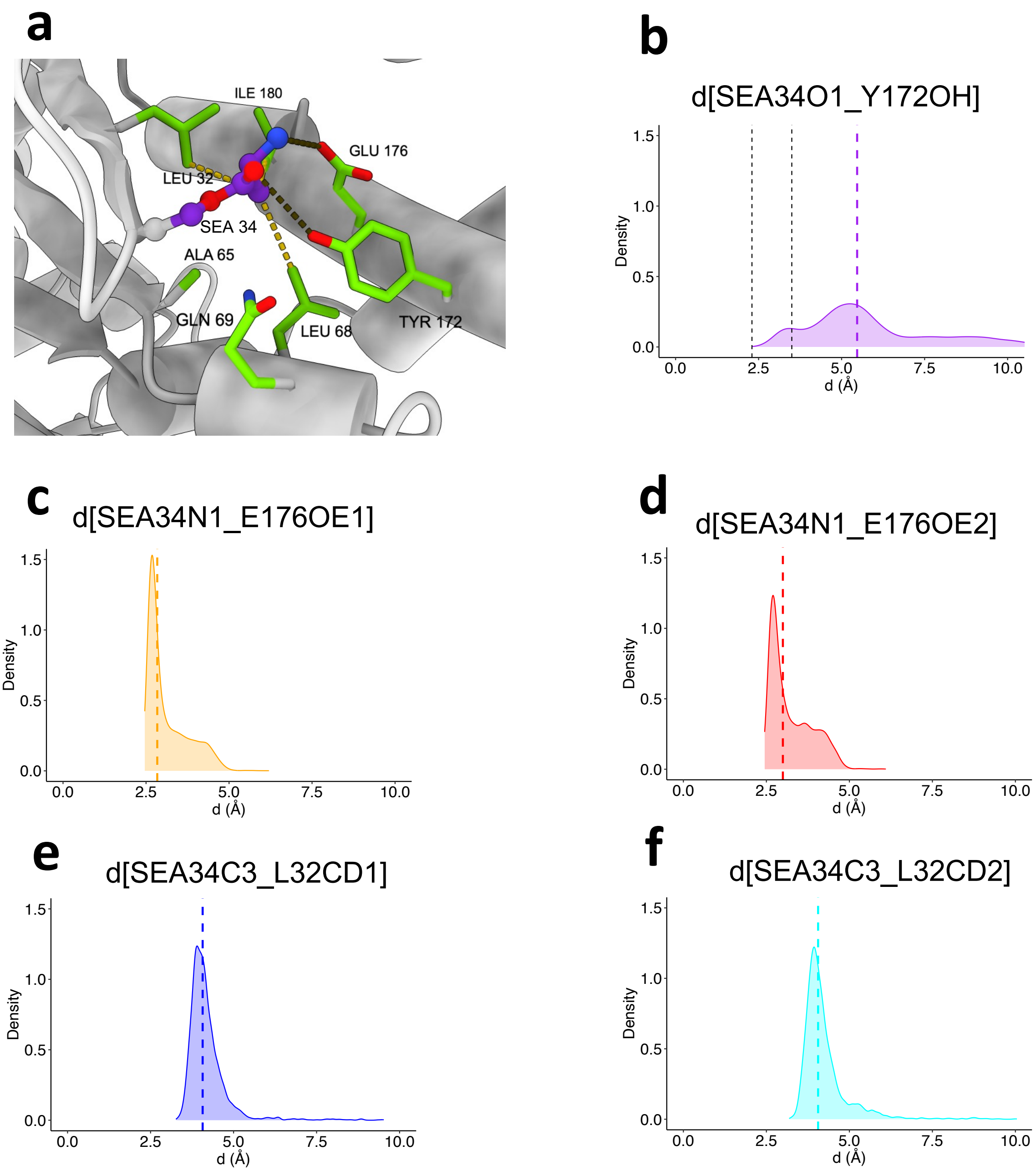

Supplementary Fig. 12: Modelling of the alanylated-enzyme intermediate. (a) Initial structure of the acyl-Ala-*Nbra*-CDPS enzyme. *Nbra*-CDPS is shown in cartoon representation coloured in grey. The acyl-Ala linked to residue S34 (SEA34) is shown in ball-and-stick representation coloured by atom-type based purple. Residues located at a distance below 4.5 Å of SEA are shown in stick representation coloured by atom-type based green. (b-f) Distribution of the distances monitored along the MD trajectory of acyl-Ala-*Nbra*-CDPS. When relevant, the limit for hydrogen bond interactions are indicated by vertical dotted lines in black. For each distribution, the median is indicated by a coloured vertical dotted line. The identity of the atoms are indicated on top of each distribution.

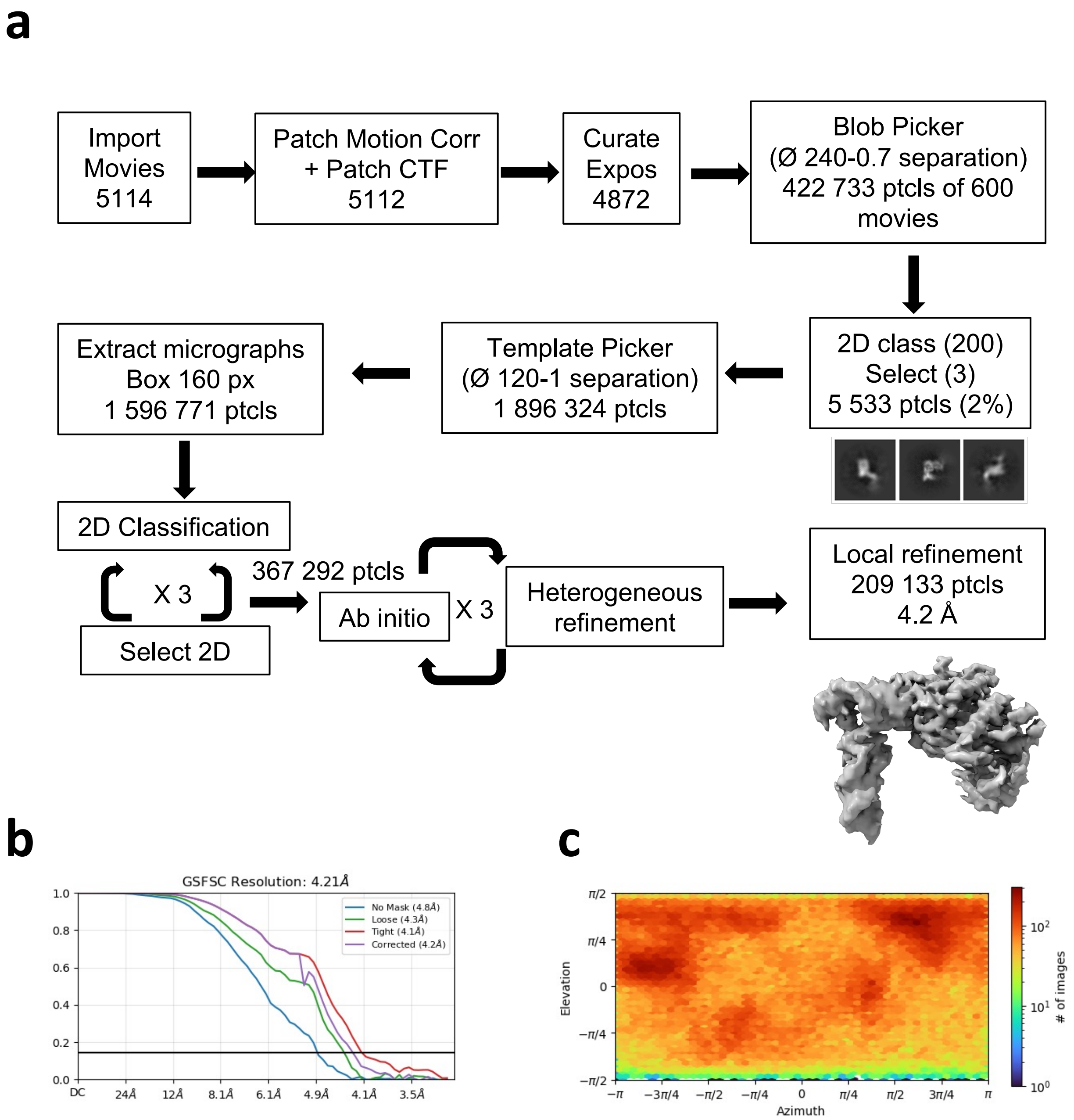

Supplementary Fig. 13: Cryo-EM data analysis of *Nbra*-CDPS^{S34A}/tRNA^{Ala} complex. (a) Single-particle cryo-EM data processing workflow in CryoSPARC. (b) Fourier Shell Correlation (FSC) plots for the final local refinement. (c) Direction distribution plot of the final particles after local refinement.

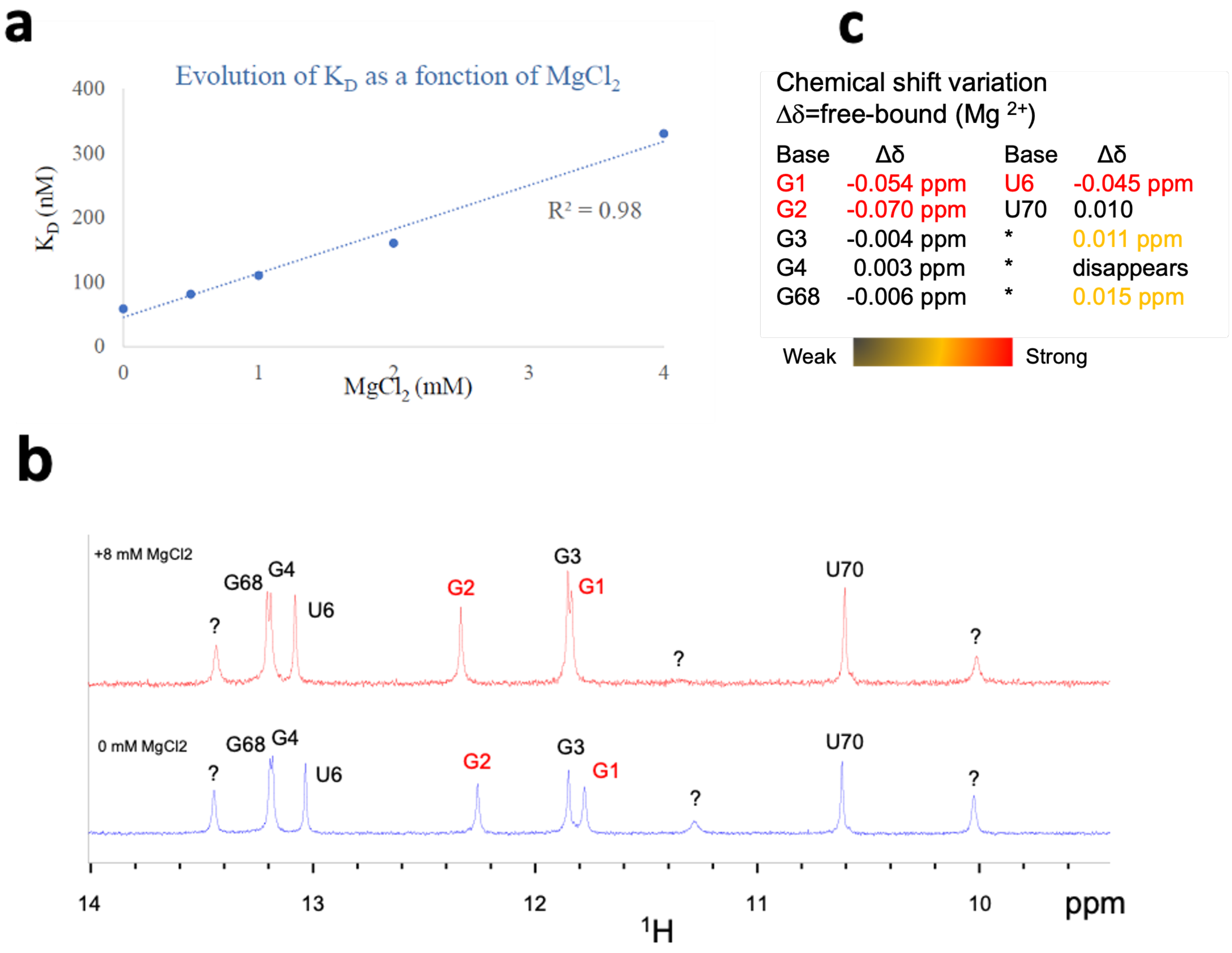

Supplementary Fig. 14: Impact of magnesium ions on *Nbra*-CDPS/miHx^{Ala} interaction. (a) Measurement of K_D by BLI between *Nbra*-CDPS$^{mono/S34A}$ and miHx^{Ala} in presence of varying concentration of $MgCl_2$. (b) Comparison of the 1D ^1H NMR spectra of the imino protons of miHx^{Ala} recorded at 950 MHz frequency in the absence of $MgCl_2$ (bottom) and in the presence of 8mM $MgCl_2$ (top), showing that the structure of the stem structure is maintained even at high $MgCl_2$ concentration. The spectra were recorded at 283K on an AVANCE Bruker 950 MHz spectrometer on miHx^{Ala} in solution in 90% H2O/10% D_2O containing 100 mM phosphate sodium, 100 mM NaCl, 5% glycerol pH 7.3, 1 mM DTT. (c) Chemical shift variation of imino protons with or without $MgCl_2$ (8 mM) extracted from the spectra shown in panel (b). The largest chemical shift perturbations are located at the 5' end of miHx^{Ala}.

a

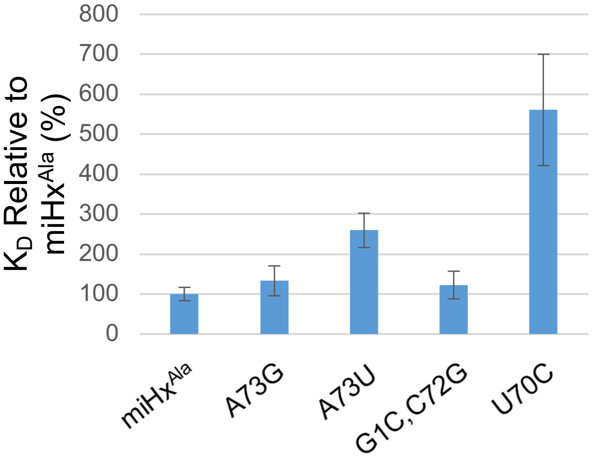

b

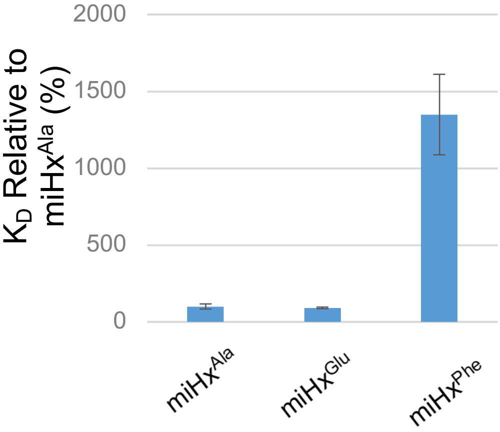

Supplementary Fig. 15: Dissociation constants K_D of *Nbra*-CDPS$^{mono/S34A}$ with various miHxs, measured by BLI. K_D values are normalized to miHx^{Ala} (set at 100%). (a) K_D values obtained with miHx^{Ala} mutants. (b) K_D values obtained with various miHxs.
